# Proteomic Snapshots of Structural Cross-Linking Rearrangements in Ca^2**+**^/Calmodulin-Dependent Kinase-1-Delta Associated with Its Regulation by ATP, Ca^2**+**^/Calmodulin, and Reduction Potential

**DOI:** 10.1021/acs.jproteome.5c00981

**Published:** 2026-03-16

**Authors:** Lutho Mbabala, Ndivhuwo O. Tshililo, Mare Vlok, Iolanda Vendrell, Roman Fischer, David L. Tabb, Catharine A. Trieber, Trixie Rae C. Adra, Michael Overduin, Sam Butterworth, Colin P. Kenyon

**Affiliations:** † South African Medical Research Council Centre for Tuberculosis Research, Division of Molecular Biology and Human Genetics, Faculty of Medicine and Health Sciences, 121470 Stellenbosch University, Cape Town 7505, South Africa; ‡ Trace Laboratories, Camdeboo Str, Belville, Cape Town 7505, South Africa; § Target Discovery Institute, Centre for Medicines Discovery, Nuffield Department of Medicine, 6396 University of Oxford, Oxford OX3 7FZ, UK; ∥ European Research Institute for the Biology of Ageing, University Medical Center of Groningen, Groningen 9713 AV, Netherlands; ⊥ Department of Biochemistry, Faculty of Medicine and Dentistry, 3158 University of Alberta, Edmonton T6G 2H7, Canada; # Division of Pharmacy and Optometry, School of Health Sciences, Manchester Academic Health Sciences Centre, 5292 University of Manchester, Manchester M13 9PL, UK

**Keywords:** phosphate and cysteine cross-linking, loop linking, kinase structural rearrangements

## Abstract

Ca^2+^/calmodulin-dependent kinase 1 delta (CaMK1δ) plays a central role in regulatory pathways associated with ATP, reduction potential, and Ca^2+^/calmodulin (CaM). Mass spectrometry (MS)-based structural proteomics incorporating FragPipe and pLink cross-link analysis was used to reveal conformation selection induced by dialysis with ATP, reducing agents, and CaM. The structural changes were mediated via cysteine and phosphate cross-linking and loop-linking of the activation loop within the C-terminal. Phosphate loop-linking was validated by β-elimination and Michael addition (BEMAD) reactions, aligning these findings with phosphoproteomics analyses of phosphorylation events. Oxidizing conditions inhibited the functionality of CaMK1δ wild-type. A novel mechanism of autoinhibition via cysteine cross-linking between the activation loop (αT) and C-terminal (αI) helices was identified. The microenvironment associated with CaMK1δ, including ATP availability, CaM concentration, and reduction potential, modulates the structural rearrangements underlying autophosphorylation. Phosphoproteomics, cysteine and phosphate cross-linking MS, and structural molecular modeling were used to describe kinase activation, allowing the activation of regulatory kinases to be reevaluated. We propose that regulatory kinases respond to an array of kinase family-specific distinct second messengers which can be studied using this multiomics framework, giving significant new insights into PTMs as well as the associated protein structure rearrangements.

## Graphical abstract

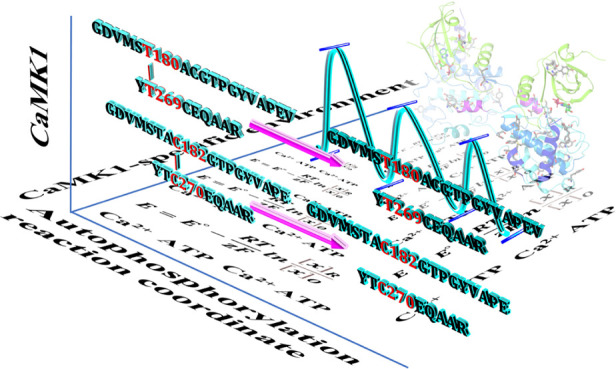

## Introduction

Phosphorylation is one of the most common and structurally transformative post-translational modifications of interest in proteomics. It not only regulates kinase activity but also protein conformation, stability, and protein–protein interactions.^1–4^ The covalent linkage of the phosphate group introduces bulk, polarity, and negative charge, resulting in often profound structural rearrangements. The convergence of phosphorylation, autophosphorylation, and tyrosine phosphorylation is critical in understanding the dynamics of kinase signaling.

Phosphorylation influences the local electrostatic environment of a protein, often initiating secondary and tertiary structural changes.^4^ This occurs by 1) disruption of hydrogen bonds resulting in destabilizing or stabilizing specific folds, 2) creation or elimination of binding motifs, and 3) inducing local disorder or order, especially in intrinsically disordered regions (IDRs). A common example is the activation loop that shifts from a flexible, disordered, and inhibitory conformation to an ordered state that aligns catalytic residues for substrate binding.^5,6^

Autophosphorylation is a mechanism for kinases to self-modulate by inducing structural changes crucial for specific activation and regulation.^7–11^ It often occurs on activation loops and regulatory domains: 1) *trans*-autophosphorylation—the extension of the activation loop, stabilizing an extended conformation that allows simultaneous activation of the interacting kinases, 2) *cis*-autophosphorylation—the dual-specificity tyrosine-regulated kinase class 2 (DYRK2) adopts an intermediate conformation that autophosphorylates during translation.^12,13^ This intermediate is short-lived, serving only to autophosphorylate a tyrosine residue and therefore differs from the native fold, which phosphorylates substrate peptides exclusively on serines and threonines.^12,14,15^ Furthermore, major structural rearrangement occurs in receptor tyrosine kinases where autophosphorylation of the intracellular C-terminal results in exposure or creation of docking motifs, recruiting proteins possessing the src homology 2 (SH2)- or phosphotyrosine binding (TB)-domain.^16^ This structural reorganization transforms the receptor from a passive transmembrane scaffold into a signaling hub with multiple protein–protein interaction interfaces.

The aromatic ring and side-chain positioning of tyrosine residues enables phosphorylation to have wide-reaching effects on protein structure. Phosphotyrosines (pTyr) achieve this by: 1) serving as docking sites for signaling complexes via protein binding domains (SH- and PTB-domains), 2) highly specific binding interfaces compared to Ser/Thr, and 3) taking part in long-range conformational shifts via allosteric coupling.^1^ Due to the structural and functional importance of pTyr, its production and recognition mechanisms are highly correlated and conserved.^17^

These three related phosphorylation processes highlight that phosphorylation is not merely a biochemical switch but also influences the structural architecture associated with kinase cellular function. Furthermore, the occurrence of other regulatory motifs on the same polypeptide chain influences how these phosphorylation events are executed. Of interest in this study is CaMK1δ, which serves as a paradigm for the larger family, the calmodulin-dependent protein kinase 1 family (CAMK1), comprising four members, CaMK1α, CaMK1β/Pnck, CaMK1γ/CLICK3, and CaMK1δ/CKliK (Figure 1).^18^

**Figure 1:**
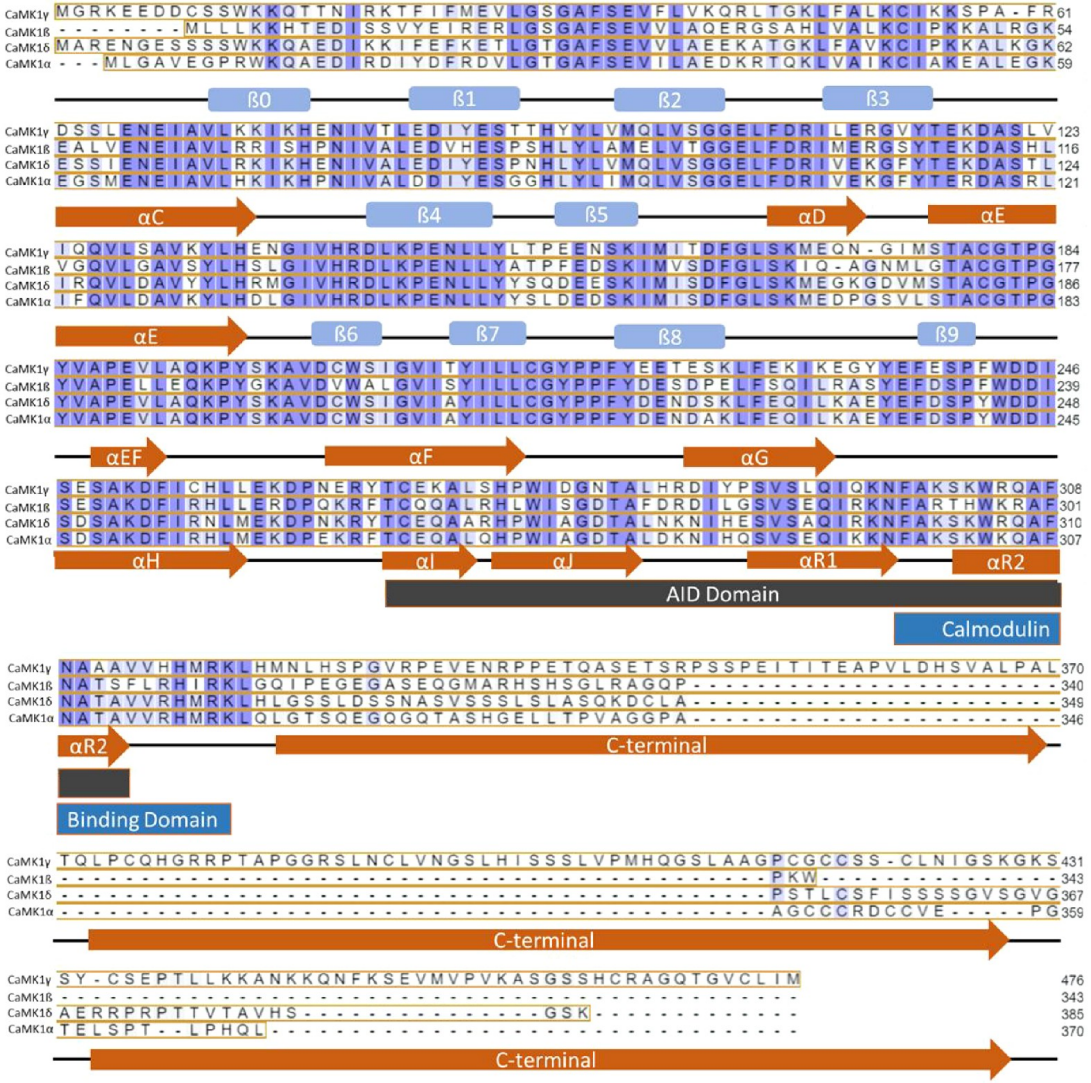
Sequence alignment of CaMK1 subfamily members. Most sequence variation is found in the C-terminal. Conserved residues are shaded in purple, with the darkest shade indicating highly conserved residues and the lighter shades indicating conserved functionality. Conservation of secondary structural motifs is shown in red arrows for alpha helices and blue squares for beta-sheets. Sequences aligned on UniProt using Clustal Omega and secondary structure prediction done on JPred 4.^19^ CaMK1 UniProt accession numbers: CaMK1-α, Q14012; CaMK1-β, Q6P2M8; CaMK1-δ, Q8IU85; CaMK1-γ, Q96NX5.

These have comparable regulation in the catalytic domain, differing primarily in the C-terminal regulatory elements that are largely responsible for each family member’s distinct functionality. CaMK1δ is responsible for activating CREB-dependent gene transcription upon calcium influx, and it regulates calcium-mediated granulocyte function and respiratory burst. CaMK1δ also promotes dendritic growth of the hippocampal neurons.^20–22^ Calmodulin (CaM) is a ubiquitous intracellular calcium (Ca^2+^) sensor protein essential for rapid and coordinated responses of a variety of enzymes, channels, and receptors to both local and global Ca^2+^ fluxes.^23^ CaMK1δ requires calmodulin binding and autophosphorylation for its optimal function. Adjacent to the autophosphorylation site is a catalytic cysteine that is sensitive to redox potential changes and plays a role in regulation.^24^ Intuitively, thiol–disulfide exchange is affected by redox signaling in the cell, especially in the role played by H_2_O_2_. Peroxide functioning as a second messenger, activating signaling cascades of downstream proteins, allows for modulation of activity and the engagement of redox regulation.^25^

CaMK1α is directly inhibited by S-glutathionylation via the catalytic cysteine, C179, whereas the mutant C179V showed no sensitivity to oxidation and maintained its activity.^26^ The inactivation of CaMK1α by *S*-glutathionylation is alleviated by the addition of dithiothreitol (DTT). Seminal work by Byrne and coworkers investigated the conserved redox control of Ser/Thr protein kinases by reversible cysteine oxidation of Aurora A and other kinases.^24^ It was shown that kinase activity is blocked by oxidation of the conserved catalytic cysteine, which corresponds to C182 in CaMK1δ, without affecting the binding of ATP. The activity was rescued by the addition of DTT. These interactions result in a protein that responds to three input signals: ATP, Ca^2+^/calmodulin, and redox potential, which are interpreted into array output messages related to specific functions.

Though crystal structures have played an important role in annotating protein architecture, they often fail to capture transient structural changes due to post-translational modifications (PTMs), especially as flexible or disordered domains are not compatible with the static nature of crystallography, resulting in low or poor electron density. On the contrary, mass spectrometry (MS) enables the observation of transient structural features.^27^ Major contributions from cross-linking mass spectrometry (XL-MS) have brought about an understanding of protein intermediate state interactions during conformational changes.^28^ These intermediate states are trapped by these covalently linked residues within defined distances. Considering the significant conformational changes exerted by phosphorylation, it can be conceptualized as a natural cross-linker, where a phosphate group simultaneously bridges residues, forming loops and driving protein folding and functionality. These phosphate bridges might be stabilized as the phosphorane intermediate during an addition–elimination reaction.^29^ Central to this investigation was the development of techniques to identify and validate the presence of phosphate and cysteine cross-linking and loop-linking. This was done in part using the pLink suite of software.^30^ pLink is a proteomics software tool used for identifying and analyzing cross-linking peptides from mass spectrometry experiments, assisting with the mapping of intra- and intermolecular interactions in proteins. Due to phosphate’s low ionizability in the mass spectrometer, an enrichment can be conducted using chemically targeted identification (CTID) approaches.^31^ The base-dependent reaction removes phosphate, leaving an unsaturated α,β-unsaturated α,β-carbonyl at the site of phosphorylation, replacing it with a signature “tagged” fragment dissociation.^32–34^ We therefore set out to demonstrate the presence of phosphate cross-linking and loop-linking by searching for mass shifts corresponding to the mass of the dissociated cross-linker on one of the peptides involved in cross-linking. The CaMK1δ protein was recombinantly expressed and purified from *E. coli* expression system and was dialyzed under a number of conditions of ATP, Ca^2+^/CaM, and reduction potential to “trap” the protein in an array of functional forms defined by the activation environment. As the structural changes within the protein are significant, we also determined the role of disulfide bond exchange in the structural rearrangements by varying the reduction potential during dialysis. Having demonstrated the presence of conserved sites for phosphorylation, we propose a mechanism whereby this multidomain phosphorylation might be achieved via tyrosine phosphate intermediates using molecular modeling.

## Materials and Methods

### Purification and Selection for Structural Rearrangement

The *E. coli* BL21 (DE3) strain was used for CaMK1δ expression and subsequent purification using Ni^2+^-IMAC and His-tag. The list of conformations selected for using various mixtures of DTT, ATP, and CaM were: Monomer (+DTT −ATP −CaM), Phosphate dimer (+DTT +ATP −CaM), Phosphate-Cysteine dimer (−DTT +ATP −CaM), and Phosphate-Cysteine dimer with Calmodulin (−DTT +ATP +CaM). All the buffers consisted of the same base constituents: 50 mM HEPES, 0.02% NaN_3_, 500 mM NaCl, and pH 7.5, and dialysis was for 48 h at 4 °C. To the respective solutions, ATP was added to a final concentration of 1 mM, DTT at 0.5 mM, and calmodulin at double the concentration of CaMK1δ (Supporting Information, Figure S10).

### Tryptic Digestion and β-Elimination and Michael’s Addition (BEMAD)

BEMAD was used to complement pLink findings of phosphate cross-linking and loop linking.^30^ This was done by breaking the phosphate cross-links and loops across the protein using the aminoethylcysteine (AEC) modification. The resulting peptides were analyzed using FragPipe (https://fragpipe.nesvilab.org/) software.^35^ FragPipe performed a semitryptic search to reflect that the analysis was done on a purified protein.

Tryptic digestion was performed using MagReSyn HILIC beads (hydrophilic interaction chromatography) following the manufacturer’s protocol (ReSyn Biosciences, SA). A denaturation step consisted of resuspending 50 μg of sample in 50 μL of 8 M Urea, 1% SDS, 5 mM TCEP, pH 7.8 HEPES buffer. Samples were treated with *S*-methylmethanethiosulfonate (MMTS) (Merck, Germany). Peptides derived from the four dialysis buffers were divided into two. The first half was analyzed for phosphate cross-linking using the pLink software. The second half was treated with β-elimination as a complementary approach for detecting phosphate cross-linking. All solutions were made in doubly deionized water at 18 MΩ from the Milli-Q system (Millipore Corp., USA). Peptides were bound onto an in-house C18 “ZipTip” (C18 membrane matrix, Merck, Germany) with 50% methanol. For the BEMAD treatment, barium hydroxide (Ba­(OH)_2_, 90 mM) in 0.005% v/v trifluoroacetic acid (TFA) (Merck, Germany) was flushed 10 times over the C18 ZipTip containing the peptides. The tips were enclosed in a 1.5 mL centrifuge tube for 1 h at 60 °C in contact with the Ba­(OH)_2_ solution below and above the C18 bed. The matrix was washed 10 times with 50 μL of 90 mM AEC (Merck, Germany) and allowed to stand for 30 min at room temperature. Peptides were then eluted with 50% acetonitrile, dried using a SpeedVac (Eppendorf, Germany), and stored at −20 °C until analyzed with LC/MS. A general schematic of the workflow is shown in Figure 2.

**Figure 2:**
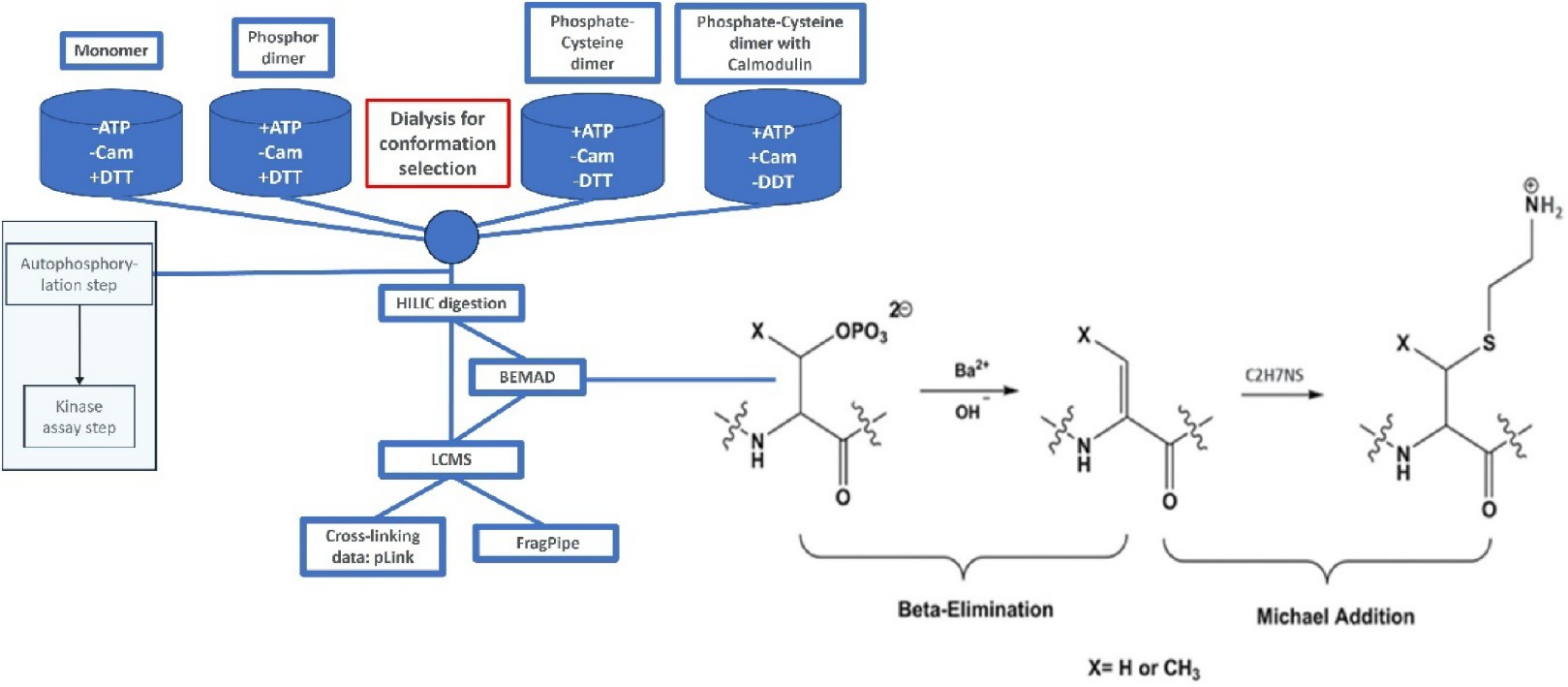
Workflow for conformational identification to characterize structural changes in CaMK1δ induced by autophosphorylation and calmodulin binding via mass spectrometry. Postpurification from nickel immobilized metal affinity chromatography (IMAC), size exclusion chromatography, and conformation selection induced during dialysis, the proteins were prepared for analysis by mass spectrometry. The different dialysis buffers used to enrich for specific conformations: 1) Monomer +DTT −ATP −CaM, 2) Phosphate dimer +DTT +ATP −CaM, 3) Phosphate-Cysteine dimer −DTT +ATP −CaM, and 4) Phosphate-Cysteine dimer with Calmodulin −DTT +ATP +CaM. Proteins were digested with trypsin on HILIC beads. Each sample was divided in two; one-half was analyzed using a standard phosphate search, and the other half was treated with 90 mM Ba­(OH)_2_ and 2-aminoethanethiol (β-elimination and Michael’s addition, BEMAD) for tracking the phosphorylated sites, especially the phosphate cross-linking sites. Autophosphorylation and enzyme activity were determined.

### Mass Spectrometry Data Acquisition and Analysis

For the LC/MS, a total of 125 ng of peptide was dissolved in 2% (v/v) acetonitrile, containing 0.1% (v/v) formic acid in nanopure water, and 1 μL was injected into the LC-MS. An Orbitrap Fusion Lumos mass spectrometer was used for analysis (Thermo Fisher Scientific) connected to a Thermo Scientific Ultimate 3000 (RSLC nano System) (Thermo Fisher Scientific). Peptides were loaded onto a PepMap C18 trap column (300 μm × 5 mm, 5 μm particle size, Thermo Fisher) and separated on a 50 cm EasySpray column (ES903, Thermo Fisher) using a 60 min linear gradient going from 2% to 35% buffer B (A: 5% DMSO, 0.1% formic acid; B: 5% DMSO, 0.1% formic acid in acetonitrile) at a flow rate of ∼250 nL/min. Data were acquired in data-dependent mode acquisition (DDA), with the advanced peak detection (APD) feature enabled. Full scans (MS1) were acquired in the Orbitrap at 120k resolution over an *m*/*z* range of 400–1500, AGC target of 4e5, S-lens RF of 30, maximum injection time of 50 ms, and a dynamic exclusion of 3 s. Fragment ion spectra (MS/MS) were obtained in the Orbitrap at 30k resolution with a Quad isolation window of 1.6, 5e5 AGC target, and a maximum injection time of 54 ms, with HCD activation and 30% collision (Figure 3).

**Figure 3:**
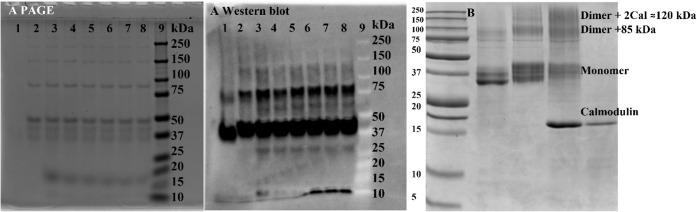
Nondenaturing PAGE and corresponding Western blot analysis of CaMK1δ. A) Nondenaturing PAGE and Western blot analysis of CaMK1δ, confirming the presence of multiple oligomeric forms. The observed bands correspond to the monomer (∼38 kDa), dimer (∼75 kDa), and tetramer (∼124 kDa), indicating that CaMK1δ may exist in various oligomeric states. Lanes: 1, CaMK1δ monomer; 2, monomer plus ATP; 3–8, monomer plus ATP plus Calmodulin; 9, molecular weight marker. B) Image Lab band identification arising from dialysis reactions with/without ATP and calmodulin (Table SI 1). Lane 1 is the molecular weight marker. Lanes: 2, dialysis without ATP; 3, dialysis supplemented with 1 mM ATP; 4 and 5, both large peaks obtained from size exclusion of the dialysis supplemented with ATP and calmodulin. The size exclusion was run in HEPES supplemented with 1 mM CaCl_2_ to avoid calcium phosphate precipitate and reduce calmodulin dissociation. Conformation descriptions for the gel are based on theoretical molecular weights. Three distinct domains were obtained in each lane. The higher molecular weight region >100 kDa and the dimer region at 75–80 kDa on the addition of ATP and calmodulin.

The mass spectrometry raw data included in this paper have been deposited to the ProteomeXchange Consortium via the MassIVE partner repository with the data set identifier PXD069258.^36^

All cross-link data were analyzed using pLink 2.0.^30^ Since CaMK1δ was expressed in *E. coli*, spectra were searched against the full *E. coli* background proteome downloaded from UniProt. Peptide identification was performed using a false discovery rate (FDR) ≤ 5%, enzyme trypsin, modifications carbamidomethyl [C], oxidation [M], phosphorylation [S, T], linker specification disulfide bond (SS), 3 missed cleavages, precursor tolerance ±10 ppm, and fragment tolerance ±10 ppm. The following phosphate linkers were explored: PO, PO_2_, PO_3_, PO_4_, HPO_2_, HPO_3_, and HPO_4_. Only PO_2_ gave the relevant data.

FragPipe was used in analyzing the BEMAD (β-Elimination followed by Michael Addition) reactions, enabling the detection of the counterpart species at the same modification sites following phosphate cross-link and loop-link removal. In this process, the phosphate group was replaced by a stable placeholder, the 2-aminoethanethiol (AEC) moiety, resulting in a +59.019 Da mass shift via the Michael addition reaction. Additionally, on the opposing counterpart site, a −18.011 Da was observed. The loss of 18 Da could be either a dehydration or the loss of phosphoric acid (98 = 18 + 80), confirming successful phosphate elimination and subsequent modification.

### Analysis of Redox-Regulated Cysteine Cross-Linking Using Nondenaturing PAGE and MS

Cysteine cross-linking was investigated by using the pLink suite software. The cysteine cross-link results in a −2 Da change. The data processing using pLink also allows for the use of pLabel, a visualization and annotation tool within the pFind proteomics software suite that is used to manually inspect, validate, and interpret tandem mass spectrometry (MS/MS) spectra. Its primary function is to annotate fragment ions by mapping observed peaks in an MS/MS spectrum to theoretical fragments derived from a proposed peptide sequence. Protein from purification was prepared for the kinase assay in the autophosphorylation step, supplemented with H_2_O_2_, GSSG, and both H_2_O_2_ and GSSG. A volume of 3 μL was added to 300 μL (±42 nM CaMK1δ concentration) of the kinase assay. The remaining volume of the autophosphorylation reactions was loaded on nondenaturing PAGE gels for band conformation analysis using mass spectrometry. Gel bands were excised, and reduced cysteines were alkylated before extracting peptides. The bands were trypsin-digested and alkylated without the first reduction, so we could chemically mark the ones that were free cysteines.

The protocols for the autophosphorylation reaction and kinase enzyme assay are outlined in the Supporting Information.

## Results and Discussion

### CaMK1δ Regulatory Molecules Crucial in Conformation Selection

To determine whether the higher molecular weight species observed during CaMK1δ purification were due to coeluting proteins or intrinsic oligomerization, Western blot analysis was performed using an anti-CaMK1δ antibody (ABCAM, UK). All PAGE was performed under nondenaturing conditions. The analysis revealed distinct bands corresponding to the monomer (∼38 kDa), dimer (∼75 kDa), and tetramer (∼100–150 kDa), confirming that CaMK1δ exists in multiple oligomeric forms rather than being contaminated by copurified proteins. These results suggest that CaMK1δ undergoes self-association, based on the availability of ATP and calmodulin, forming part of the autophosphorylation process. The effect of ATP and calmodulin on the oligomerization of CaMK1δ was assessed. The presence of ATP and/or calmodulin also affected the band positions, indicating structural rearrangement especially in the monomeric forms. These data indicate significant structural rearrangement of the CaMK1δ associated with the presence or absence of ATP and/or calmodulin.

### Identification of Phosphate Loop-Linking and Cross-linking Using Mass Spectrometry

Having established the addition of ATP and calmodulin during dialysis affected the structural forms of CaMK1δ, we carried out the dialysis of CaMK1δ in the presence and/or absence of ATP, calmodulin, and DTT, to establish their role in generating the atypical mass shifts visualized by native PAGE. We set out to establish the role of phosphorylation in the formation of the different structural forms associated with the structural rearrangement, extending the analysis to demonstrate the possible presence or absence of a phosphate cross-linking, loop-linking, and single-site phosphorylation within the protein during the conformational changes. This hypothesis required validation as there is no documented evidence of phosphate being implicated in the structural rearrangement of proteins via cross-linking or loop-linking intermediates of proteins.

The use of pLink software identified cross-linking peptides at ≤0.1% of the total number of peptides, while the loop-linking peptides had a 10-fold higher frequency (Table S2A and B). Table S2A and B compare results from spectra searched against 100 *E. coli* proteins (A) to the whole proteome (B). We define cross-linking as a phosphate group spanning two distinct peptide fragments and loop-linking, a phosphate group cross-link, within a single peptide. The proposed mechanisms associated with phosphate cross-linking and loop-linking are outlined in Figure S1. Interestingly, loop-linking occurred mainly in the activation loop (Ser179 and Thr180, DVM**ST**ACGTPGYVAPEVLAQKPYSK or between Thr180 and Thr184, MS**T**ACG**T**PG) and in the C-terminal region (Ser351 and Thr352, DCLAP**ST**LCSFISSSSGVSGVGAER), Table S3A (100 *E. coli*proteins) and Table S3B (*E. coli*proteome). The loop-linking occurred primarily under dialysis conditions intended for the formation of dimers (in the absence of DTT). These data suggest conserved functionality in the protein with these domains having a role in conformation selection. By way of example, the pLink software was used to annotate loop-linking and cross-linking peptides. The proline effect, wherein the fragment ions on the N-terminal of the proline residue show higher intensity, was also identified, with examples shown in Figure 4.^37^

**Figure 4:**
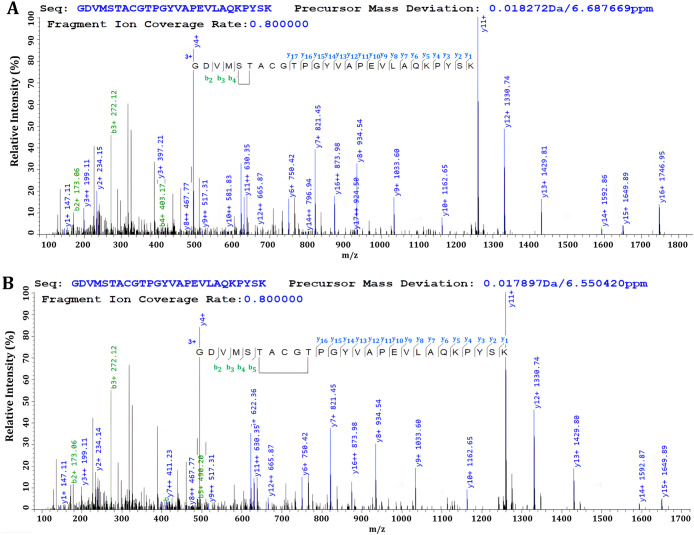
Example of pLink-annotated phosphate loop-linking spectra within the activation loop. The phosphate loops have been annotated between A) S179 and T180 and B) T180 and T184.

The dehydration taking place upon the cross-linking results in the phosphate losing an OH when attaching to the side chain of serine or threonine. Thus, the mass of the linker is calculated to be 63.97 Da for HPO_2_ and 62.96 Da for PO_2_, as shown in Figure S1. To validate the presence of phosphate cross-linking and loop-linking, further analysis using FragPipe tested for +62.9 (O=P—O) and +63.9 (HO—P=O) mass shifts. These were taken to be indicative of the loop-link formation. In this hairpin form, breaking a side chain in the cyclic structure does not give a fragment ion unless another fragmentation occurs. Therefore, the b- and y-ions outside the cross-link should still be visible, and the fragment ions associated with the loop will occur less frequently.

### Validation of Phosphate Loop-Linking and Cross-linking by Linker Mass Identification

In an attempt to validate the presence of loop-linking and cross-linking, a comparative analysis was carried out on the two distinct experiments. The phosphorylation PTMs were identified before and after BEMAD treatment using the pLink suite of software. These data sets were then also subjected to analysis using the FragPipe computational platform to establish the presence of the +62.9 (O=P—O) and/or the +63.9 (HO—P=O) mass shift. Most identifications represented peptides in the activation loop and the C-terminal region (Table 1). In Table 1, the phosphate (pre-BEMAD) and phosphate (post-BEMAD) loop data are shown. In gray, the low peptide frequencies in the phosphate pre-BEMAD are indicative of the presence of phosphate cross-linking as the FragPipe analysis is not capable of identifying the three-way phosphate cross-link (Figure S1). Similar data were obtained when doing the search against an *E. coli* database comprising 100 proteins; however, the relative frequencies were much higher (Table S14). The phosphate arises as a result of the BEMAD reaction creating monolink phosphate arising from the β-elimination from the opposing phosphate-linked peptide. Therefore, on BEMAD treatment, the linker is identified by FragPipe, being indicative of the remaining loop-link after the β-elimination. This was found primarily in the activation loop. A high peptide frequency was observed at the C-terminus, also suggesting loop-linking. In the activation loop (gray), the post-BEMAD PTMs mass is indicative of the dissociation of the cross-linking taking place leaving the residual loop-link left of the peptide pair which has not taken up the AEC modification (Table S4). On the other hand, the C-terminal region (in blue) has more loop-linking in the phosphate PTMs pre-BEMAD section, which indicates true intrapeptide loop-linking. This is also demonstrated in the AEC modification post-BEMAD treatment (Table S4).

**Table 1:**
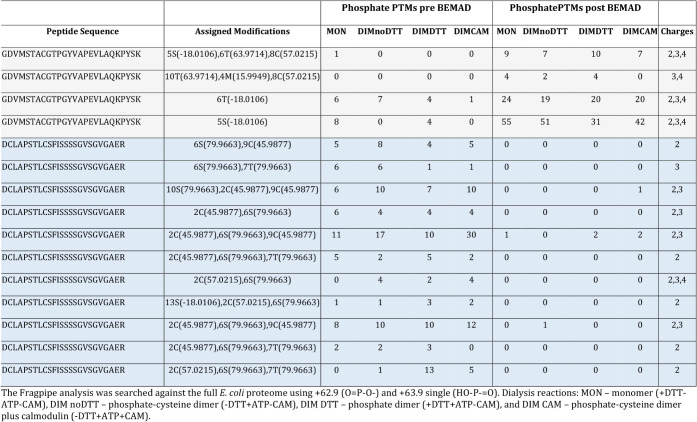
FragPipe Analysis of the Mass Shifts Indicative of Phosphate Loop-Linking and Cross-Linking

### Phosphate Cross-Linking Validation via BEMAD

BEMAD was used as a complementary method to identify cross-linking and loop-linking. The BEMAD reaction replaces the phosphate group with a stable placeholder, the 2-AEC moiety, resulting in a +59.019 Da mass shift via the Michael addition reaction. Additionally, a mass shift of −18 Da may also be observed at the same sites. The loss of 18 Da could be either a dehydration or a loss of phosphoric acid (−98). The fact that both mass shifts are detectable suggests the detection of both sides of the cross-linked peptide following the BEMAD separation.

For some peptides, all three reinforcing chemical modifications, phosphorylation, AEC, and the loss of phosphoric acid, were present in Table S4. The enumeration tables of single-site phosphorylations, all −18 Da (loss) sites, only occurring under BEMAD conditions, and pre- or post-BEMAD treatment are outlined in Tables S5–S7. Examples of the fragmentation coverage were also obtained in the samples arising from the BEMAD analysis Figures S2 and S3. The activation loop is predominantly defined by the y-ion series, and there was an oxidation of methionine and an AEC modification on the serine (Figure S2A). The spectrum contains minimal misidentified peaks, improving confidence in the assignment. The peptide in Figure S2B is part of the αC helix and demonstrates strong fragmentation coverage for both b- and y-ions, with an AEC on the fourth serine.

### Effect of CaMK1δ Conformation Selection by Dialysis on Cysteine Cross-Linking

Having established domain-specific phosphorylation sites dependent on the dialysis environment, the investigation was extended to the role of cysteine cross-linking in the structure of CaMK1δ. CaMK1δ contains seven cysteine residues; however, two cysteine sites of cross-linking were identified forming between the activation loop and the autoinhibitory domain, namely Cys182-Cys270 (monomer conformation) and C270–C270 (dimer conformation). The activation loop Cys182 cross-link to the αI-helix Cys270 was found in all dialysis conditions. The pLink data are summarized in Table S8. Functionally, the dominant cysteine cross-linking obtained was the activation loop linked to the autoinhibitory domain housed on the αI-helix (αI-helix, Figure 5). This activation loop Cys182 (DVMSTAC182GTPGY) cross-linked with the autoinhibitory domain Cys270 (αI-helix, RYTC270EQAAR) is unprecedented and suggests inhibition via a disulfide bond, instead of the canonically accepted mechanism of hydrophobic interaction.^38^ Recent work by Bendzunas et al.^39^ explored redox regulation of AMPK-related, brain-selective kinases (BRSK1 and 2). They demonstrated catalytic activity being directly regulated via a conserved reversible cysteine bond in the activation loop and the catalytic HRD motif.^39^ In CaMK1δ, this regulation was seen between the activation loop and the autoinhibition domain. The spectra shown in Figure 5 demonstrate the activation loop (C182) cross-linked with the autoinhibition domain (C270), with a strong y-ion series annotating most of the dominant peaks in the spectra; the proline effect contributes intense fragments at three peptide bonds.

**Figure 5:**
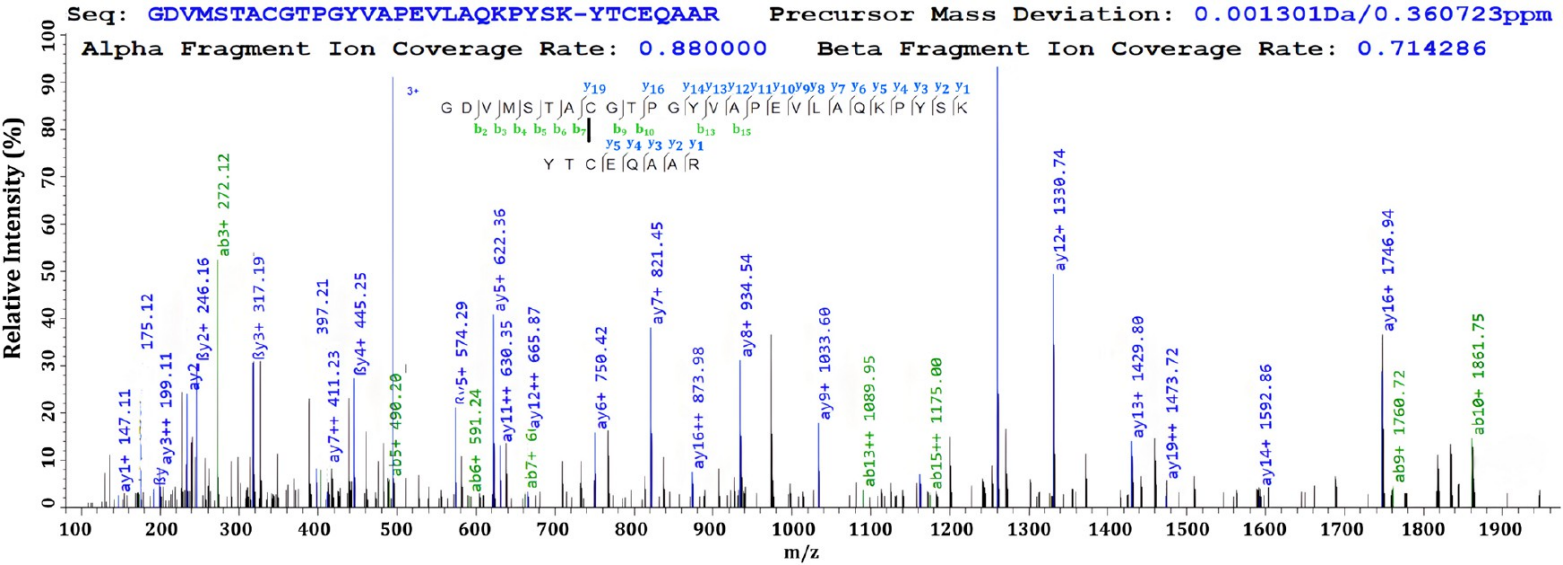
Fragmentation spectra of cross-linked peptides involving the activation loop and αI-helices. The activation loop exhibits a strong y-ion series, and αI lacks b-ions.

The pLink software suite was also used to investigate the potential presence of cystine loop linking within the protein structure. Cystine loops were found primarily in the C-terminal region involving Cys347 and Cys354 of CaMK1δ the protein, Figure 6 and Table S9. The C-terminal peptide D**C**LAPSTL**C**SFISSSSGVSGVGAER (2)­(9) was found in all the dialysis conditions and was significantly higher in the reactions designed for dimer formation, not containing DTT. The putative intramolecular cystine loop in the C-terminal region of CaMK1δ is novel as no X-ray electron density is often obtained in the C-terminal region of kinases, as a result of the intrinsically disordered domains. The functional impact of the cysteine loop formation in the intrinsically disordered C-terminal of CaMK1δ forms part of the discussion.

**Figure 6:**
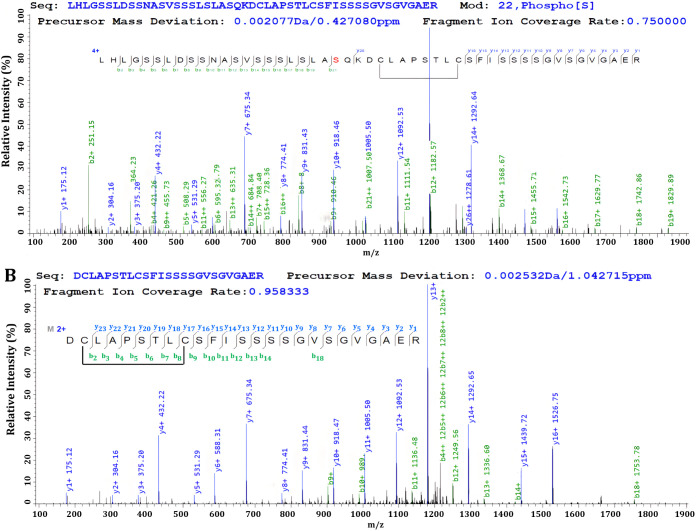
Fragmentation spectra of cysteine loop-linking spectra in the C-terminal. The N-terminus is annotated by b-ions, and the C-terminus by y-ions. The cysteine loop-link occurs centrally in the peptide, with the cysteine loop annotated, showing both b-and y-ion series.

The fragmentation spectra for the C-terminal cysteine loops are listed in Figure 6. Panel A contains the N-terminus as annotated by b-ion and the C-terminus of the peptide is annotated by the y-ions. The cysteine loop-link occurs centrally, without fragmentation occurring on the involved residues. Panel B contains the second peptide with the loop annotated, with both b- and y-ions.

### Effect of Oxidizing Conditions on Kinase Functionality and Conformation Selection

Native-PAGE gel electrophoresis was used to associate functionality with the putative phosphate and cysteine cross-linked and loop-linked states evidenced in specific bands. A total of four bands could be identified across all conditions (Figure 7). Three dominant bands appeared upon the addition of calmodulin, in the absence of DTT, namely band 4, 29.84 kDa; band 3, 33.14 kDa; and band 2, 36.60 kDa (lane 2, Figure 7A). In the presence of DTT and calmodulin, this is reduced to two bands, namely 33.14 kDa and band 1, 41.12 kDa. In all cases, the addition of DTT and calmodulin shifted the conformer equilibrium to two bands, namely 33.14 kDa and band 1, 41.12 kDa. The addition of either CaM or DTT alone gave low basal activity (Figure 7B, lanes 2 and 3). The addition of both calmodulin and DTT together, to the dialysis, was required for the maximal activation of the CaMK1δ (lane 3). In the presence of calmodulin and absence of DTT, the introduction of oxidizing conditions (both H_2_O_2_ and GSSG) resulted in the loss of the calmodulin-induced activity. Under all conditions, the presence of DTT and calmodulin induced full functionality, indicating the need for a reducing environment for disulfide bond exchange. The addition of DTT to the dialysis also appears to be required for the formation of the conformer obtained at 41.12 kDa.

**Figure 7:**
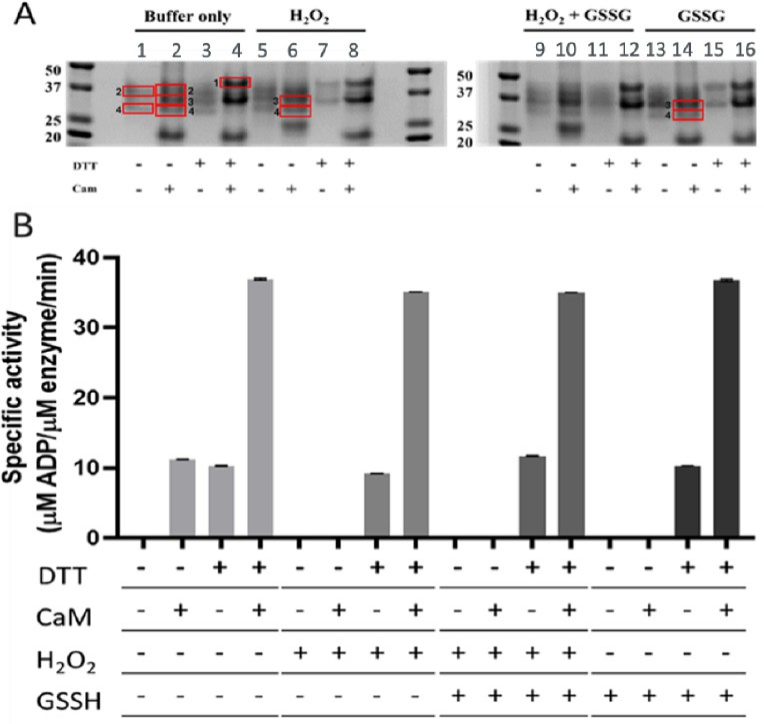
Effect of autophosphorylation and oxidizing conditions on the CaMK1δ kinase enzyme activity and conformations in the presence and absence of DTT and calmodulin. All enzyme assay reactions contained ATP. A) Bands highlighted (red boxes) were excised and digested for MS analysis. B) Kinase assay enzyme activity data. Standard autophosphorylation reactions were set up and altered by the addition of oxidizing agents H_2_O_2_ and GSSG. The kinase assay contained 42 nM of CaMK1δ. A) Autophosphorylation (20 μL) reactions were run on nondenaturing PAGE gels after 2 h of autophosphorylation. The PAGE gel and the kinase assay data are displayed in the same sequence. The whiskers represent standard deviation.

Specific bands were excised and digested with trypsin for mass spectrometry analysis, and cysteine cross-linking was enumerated (Table S10). The cysteine cross-linking peptides identified indicated that the loss of activity was due to the cross-linking between Cys182 and Cys270 (GDVMSTA**C**GTPGYVAPEVLAQKPYSK­(8), YT**C**EQAAR­(3)), within the activation loop and αI-helix, respectively. The enzyme activity associated with lane 4 was the highest when compared to the other reaction conditions. Band 1 in lane 4 also had the lowest levels of activation loop and αI-helix cross-linking, as measured by the spectral counts (Table S10). Under all conditions, bands 3 and 4 appeared to allow for structural rearrangement associated with the formation of the activation loop and αI-helix cross-linking. It is feasible that interpeptide cross-linking between two monomers might occur within the band as a result of the PAGE being run under native conditions, in the absence of any reducing agent (Figure 7). The addition of oxidizing agents enhanced cross-linking, also leading to inhibition of enzyme activity.

### Role of Cys182 and Conformational Selection on Kinase Activity

CaMK1δ was purified from three expression constructs, wild-type, and two mutant enzymes, C182A and C182A-T184V. The proteins were then dialyzed in a range of dialysis conditions: without ATP in the presence of DTT selecting for monomers (M, monomer), with ATP in the absence of DTT selecting for dimers (D, dimer), in the absence of DTT but containing calmodulin (D+C), and the fourth group containing undialyzed protein (UT, untreated protein with no dialysis, untreated for both TEV and phosphatase). The proteins were then autophosphorylated in the presence or absence of ATP and/or calmodulin (Figure S4). The three proteins were assessed for their kinase activity. The monomer reactions (enzyme in −ATP +DTT dialysis) of both wild-type and C182A had the highest activity (Figure S4A and B). Autophosphorylation in the presence of calmodulin significantly increased the activity of the wild-type enzyme. The C182A monomer was constitutively active unlike the wild-type and the C182A-T184V mutant (Figure S4B). The C182A monomer did not appear to require calmodulin for activation. The C182A-T184V mutant showed no activity for all conformations when not bound to calmodulin and low levels of activity when bound to calmodulin (Figure S4C). The dimerized CaMK1δ appeared to have lost its ability to phosphorylate the substrate (Camtide-KKALRRQETVDAL). These data served to reinforce the regulatory role identified from the proteomics data for C182 and T184.

### Tyrosine Phosphorylation as a Component in Phosphorylation

Also investigated on the samples arising from the effect of dialysis conformation selection were the levels of tyrosine phosphorylation. High spectral counts for tyrosine phosphorylation in the β4 and β5 helices, the catalytic loop, and the autoinhibitory domain, αI-helix, were obtained (Table S11): spanning β-sheets 4 and 5—Tyr88 and Tyr95; the catalytic loop—Tyr152 and Tyr153; and in the autoinhibitory domain—Tyr268. Tyrosine phosphorylation was found only in the dimer folding conditions. This was interpreted as a putative role of tyrosine as a conduit in the refolding of the CaMK1δ in response to environmental stimuli.

### Tyrosines as Conduits for Phosphate Transfer in the C-Terminal Region

Cross-linking and loop-linking were restricted to a select subset of domains, suggesting that they are driven by specific conformational changes in response to the local environment. This study offers a mechanistic explanation for the occurrence of phosphate cross-linking, loop-linking, and hyperphosphorylation, across the protein without a mediating kinase for each site. We propose that phosphorylated tyrosines act as conduits, being able to span large gaps, impacting different domains of the protein while refolding, in an ATP-driven, folding energy cascade. The phosphorylation seen in our data is therefore putatively the end point to multiple transient phosphate transfers, as part of the protein folding process associated with the regulation in response to the small-molecule messengers. The folding is therefore energy-driven along a very specific folding trajectory.

### Putative Functional Implications of Identified PTMs

The major domains for cross-linking and loop linking, both phosphate and cysteine, were found to be the activation loop, comprising the αT-helix (DVM**ST**A**C182**G**T**PG**Y**), the autoinhibitory domain (αI-helix, RYT**C270**EQAAR), and the C-terminal D**C**LAP**S**TL**C354**SFISSSSG. CaMK1δ homology models, based on CaMK1α (4FGB.pdb (apo form), 4FG8.pdb, 4FG9.pdb), were built to interpret the mass spectrometry data as the starting point for the putative regulatory domain-inhibited and activated structures by restructuring the activation loop “αT-helix” Figure S6.^40^ The “active” conformation referred to here is the physiologically relevant conformation (PRC), which may not necessarily be able to phosphorylate Camtide, the substrate enzyme activity peptide. CaMK1δ shares 82.1% sequence identity with CaMK1α excluding the disordered C-terminal; thus, it is possible that CaMK1δ will also feature the αT-helix in its activation segment as obtained in the homology model.

The CaMK1α (4FG9.pdb) structure (AA17-AA317) includes the regulatory domain helices αR1 and αR2 enabling them also to be defined in the CaMK1δ model, as seen in Figure S7. Also obtained in the CaMK1δ model of this structure is the T-loop adopting a helical shape (αT-helix) from Gly175-Thr184, comprising GDVMSTAC182GTP of the activation loop. This structure was then compared with the CaMK1δ homology model based on the structure of the *trans*-activation of the DNA-damage signaling protein kinase Chk2 (2CN5.pdb), as shown in Figure S7B.^41^ This structure is a dimer with the *trans*-activation occurring via αT-helix exchange and the rotation of the αR1 helix away from the catalytic domain (Figure S7B and C). The αT-helix, from Gly175-Thr184, comprising GDVMSTACGTP, occurs in both the CaMK1δ-modeled structure as well as the Chk2-modeled structure.^40,41^

Upon ATP binding, the primary functionality arises from the rearrangement that occurs within the αT-helix (activation loop adopting a helix shape; Figure S6A, B, and C). *Cis*-autophosphorylation is illustrated in Figure S6A and C where the αT-helix positions Thr180/184 toward Tyr198/187 within the same molecule. The extended activation loop in **B** represents *trans*-autophosophorylation where Tyr187/198 is on the adjacent kinase (Figure S7C). The role of *cis*- and *trans*-autophosphorylation is interpreted as stages in the kinase activation cascade.

The dimer in the *trans*-autophosphorylation structure occurs via an αT-helix exchange between subunits, driving the rotation of the αR1 helix away from the catalytic domain (Figure S7A and C). Strategically conserved tyrosine, arginine, and lysine residues facilitate the structural rearrangement within all serine and threonine kinases, largely through electrostatic interactions derived by the high negative charge of the phosphorylation. This raises the major question: How does the phosphorylation that occurs in the αT-helix manage to get distributed to the number of conserved functional domains in response to Ca^2+^ activation and the putative associated reduction potential? The presence of phosphate cross-linking allows a mechanism by which direct phosphate linking may transfer phosphate, in addition to the typical transfer from ATP; however, the specific mechanism and resulting selectivity are not yet understood.

The proximity of the B-αT-helix, Thr180, and Cys182, held within the A-subunit active site, to the B-subunit, B-Thr269 and B-Cys270, and the juxtaposition of Tyr198 to B-Tyr268-Thr269-Cys270 is instructive of how autoinhibition via both phosphate and cysteine cross-linking might occur (Figure 8). The cross-linking in question occurs via GDVMS**T**ACGTPGYVAPEVLAQKPYSK (Thr180)-Y**T**CEQAAR (Thr269) for the phosphate cross-linking and GDVMSTA**C**GTPGYVAPEVLAQKPYSK (Cys182)-YT**C**EQAAR (Cys270) for the cysteine cross-linking. Also contributing to the mechanism are the relative positions of Cys204 and the closely associated Ser206 on αI. For inhibition, a disulfide bond, therefore, has to form between the B-αT Cys182 and Cys270 on B-αI. The B-αT-helix, therefore, has to be drawn into close proximity with the B-αI. The transient phosphorylation of Tyr198, as defined in Figure S7C, may contribute to the formation of the *trans*-autophosphorylation dimer. The phosphoryl-transfer being facilitated between the αT-helix and Thr269 being facilitated by the phosphorylated Tyr198 (Figure 8).

**Figure 8:**
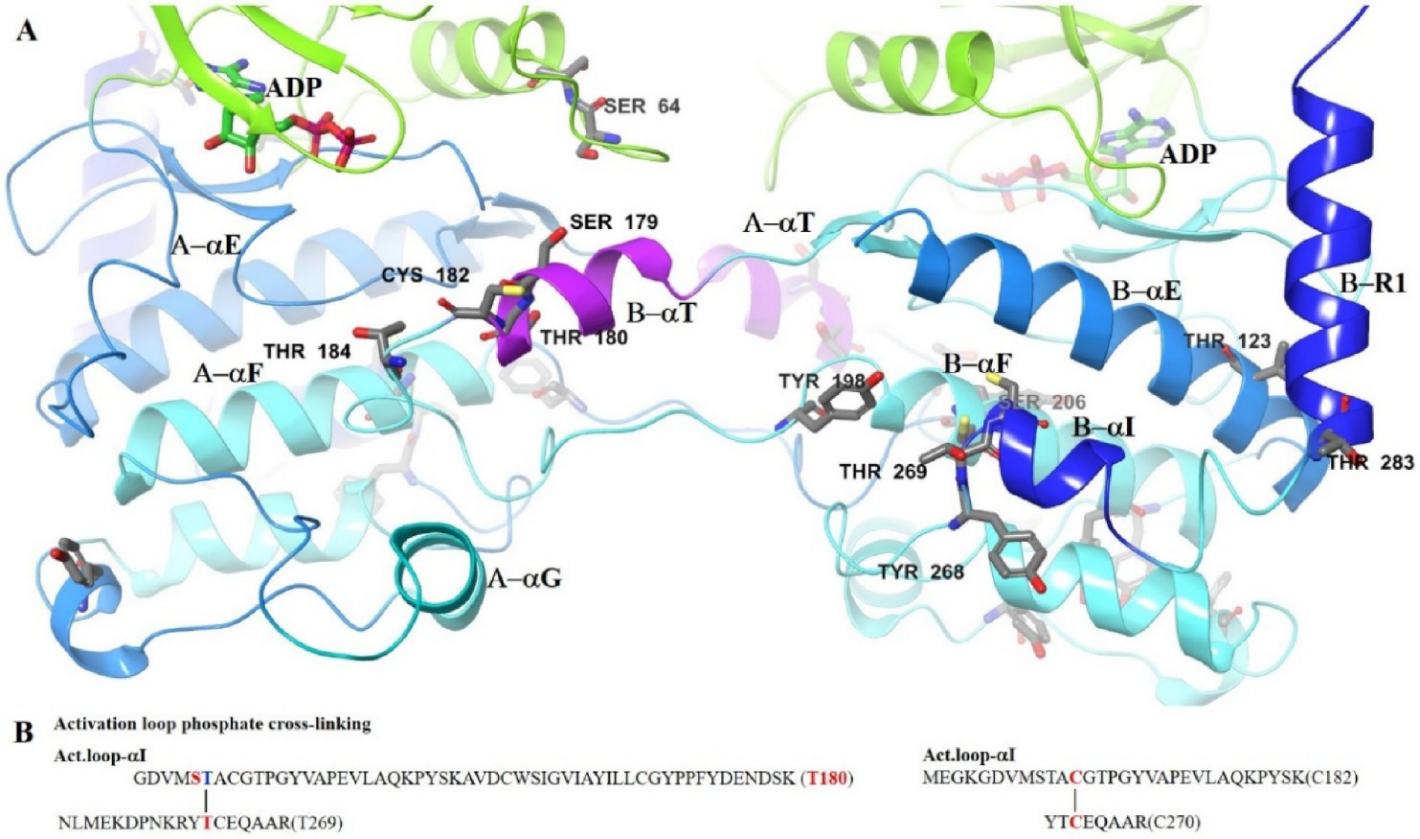
Proximity of the B-αT Thr180 and Cys182 within the A-subunit active site to B-Thr269 and Cys270 and the juxtaposition of Tyr198 to B-Tyr268-Thr269-Cys270, the identified autoinhibition site. Highlighted in (A) is the proximity of the most prevalent residues found in phosphate cross-linking (B).

The phosphorylation of Ser179, Thr180, and/or Thr184 within the αT-helix could lead to the transfer of the phosphate moiety to Tyr ,187 as the monomer (Figure S6C). In this proposed new mechanism, prior to dimerization, Tyr198 is phosphorylated from within **S**179**T**ACG**T**PG**Y187** as a “single-turnover” phosphorylation (Figure S8A). “Single-turnover” phosphorylations do not follow Michaelis–Menten kinetics in that the concentrations of kinase to substrate are at a 1:1 ratio. It is in fact plausible that as CaMK1δ responds to the change in the Ca^2+^/Mg^2+^ concentration ratio, it creates the correct binding affinities with ATP for the “initiation” cascade leading to the phosphorylation of Tyr187 and Tyr198.^42,43^ The coordination chemistry of either Ca^2+^ or Mg^2+^ defines which of Ser179, Thr180, or Thr184 are selected for phosphorylation and the stage of the reactions associated with Ca^2+^ release and reabsorption.

The phosphorylation and redox regulation of CaMK1δ therefore involve complex structural rearrangements. The rotation of pTyr187 allows proximity to Tyr198, facilitated by the interaction of Arg143 (Figure S8A and B). If the phosphate remains bonded to Thr180/184 and is transferred, bonded to Tyr187, this would localize the αT-helix to the αI-helix facilitating phosphate cross-linking between Thr180/184 and Ser269 (Thr180-PO_2_^+^-Ser269), as well as disulfide bond formation between Cys182 and Cys270, mediated by H_2_O_2_, as the intermediate (Figure S8C). The reduction of the Cys182-Cys270 disulfide, during Ca^2+^ stimulation, could be mediated via reduced glutathione (GSH), as GSH is increasingly being implicated as another small molecule second messenger, not merely an antioxidant.^44–48^ It is also conceivable that as the localized concentration of Mg^2+^ decreases (on Ca^2+^ stimulation), switching the coordination chemistry of the Thr180-PO_2_^+^-Ser269 cross-link, with the assistance of an associated H_2_O molecule, the phosphate cross-link bond is broken leaving Ser269 and Thr180-PO_3_^+^. This Thr180 phosphate may then be in close enough proximity to Cys204 to allow for the deprotonation of Cys204 to the thiolate, enabling a disulfide bond formation by disulfide bond exchange with Cys270 and thus reversing the inhibition and activating the kinase.

### Structural Rearrangement for Functionality

It is fair to assume that once activation has occurred, significant structural reorganization is required to facilitate the extensive C-terminal phosphorylation discovered. This extensive phosphorylation would itself create significant structural reorganization. This structural reorganization, in response to the change in Ca^2+^ concentration, is significant when the level of phosphorylation of the C-terminal is considered. The structural reorganization from the monomeric structure when compared with the *trans*-activation dimeric form is in itself significant (Figure S7A and B). By comparing the relative positions of αC-Glu79 and αR1-Gln296 in the monomeric structure and the *trans*-activated modeled structures of CaMK1δ, the extent of the structural reorganization required is evident (Figure 9).

**Figure 9:**
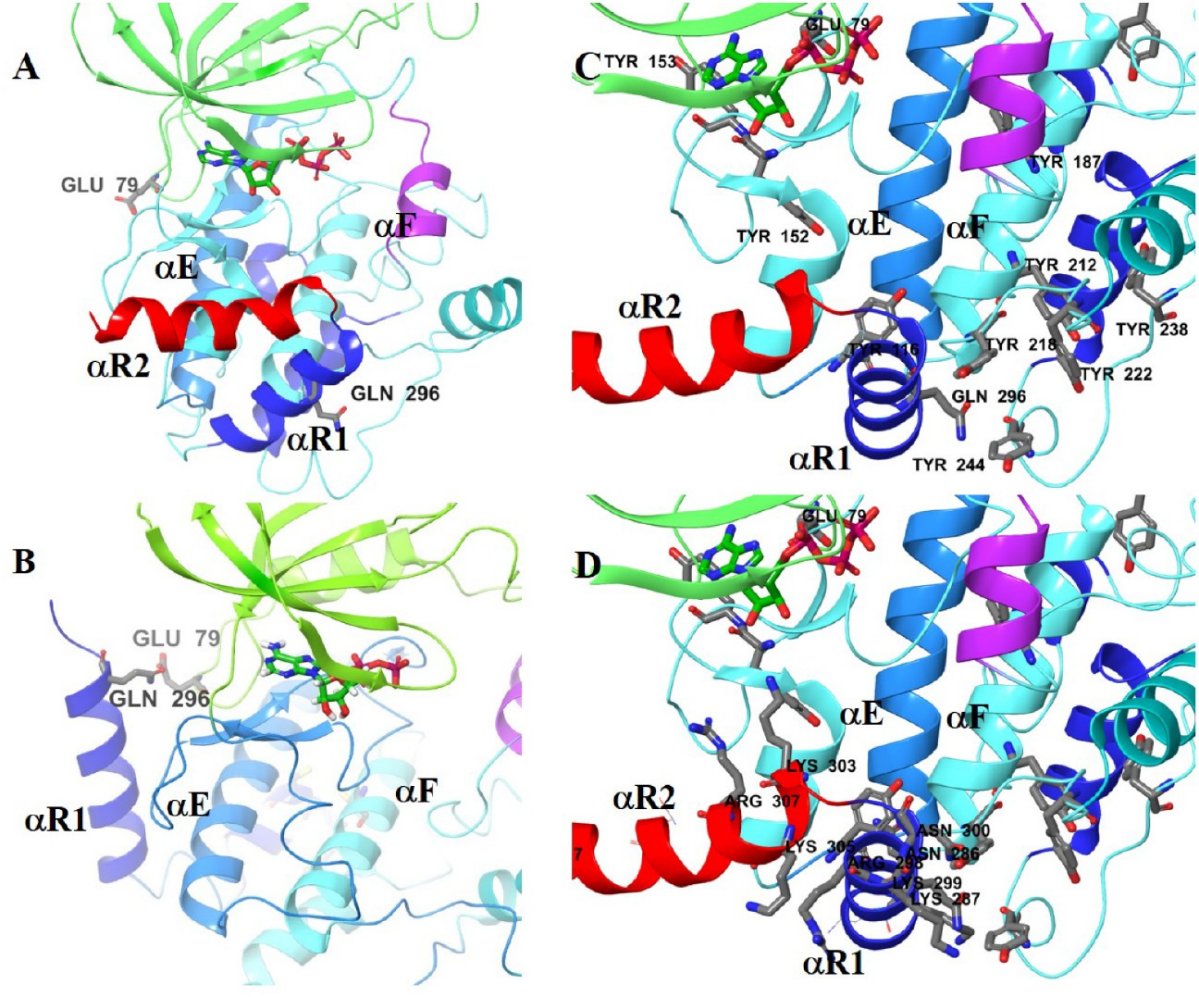
Role of electrostatic interactions resulting from the localization of PO4^2–^ on the tyrosine residues in facilitating the rotation of regulatory domain helices αR1 and αR2 away from the activation loop. A) Relative positions of αC Glu79 and αR1 Gln296 in the monomeric CaMK1δ with ATP bound (CaMK1α-based model, 4FG9.pdb). B) Relative positions of αC Glu79 and αR1 Gln296 in the *trans*-activated CaMK1δ with ATP bound (CHK2-based model, 2CN5.pdb). C) Proximity of tyrosine residues and D) proximity of associated lysine, arginine, and asparagine residues. Figures (C) and (D) are identical other than the highlighted residues to demonstrate the proximity.

The electrostatic interactions resulting from the localization of PO_4_^2–^ on the tyrosine residues would also facilitate the rotation of the regulatory domain (helices αR1 and αR2) away from the activation loop and probably Ca^2+^/calmodulin binding (Figure 9).

This implies that Tyr198 plays a crucial role not only in the autoinhibition/activation of CaMK1δ but also in directing intramolecular reorganization and C-terminal phosphorylation. The helices αF and αG and a largely disordered domain (Tyr198-Tyr244) are particularly tyrosine-rich (Figure 9C). The aim of tyrosine phosphorylation is the creation of a large domain of negative charge to facilitate the movement of entire helices to create the final structure. αR1 and αR2 provide the positive charge residues as counterions to stabilize these transitions (Figure 9D and Figure S9). Structural reorganization begins by the phosphorylation of Ser179, Thr180, and Thr184 (αT-helix), leading to the phosphorylation of Tyr187, and possibly Tyr198. These are in close proximity to Tyr133, Tyr134, Tyr212, Tyr218, Tyr222, Tyr238, Tyr244, and Tyr268 indicating a putative role of all these tyrosine side chains in the activation rearrangement. This rearrangement cascade is established by ATP-derived energy and regulated phosphoryl transfers, rather than random folding, speaking to a guided mechanism for conformational change that is not merely driven by hydrogen bonding and hydrophobic interactions.

## Conclusion

The data outlined indicated a putative role of phosphorylation and cysteine cross-linking as the source of energy associated with driving the structural rearrangements associated with the stimuli of kinases. The phosphorylation and disulfide bond profiles outlined in this investigation fall outside the current model of how protein kinases are activated by phosphorylation and the role of this phosphorylation in their functionality. In the standard model for kinase functionality, for a substantial subset of the protein kinases, catalytic activity is dependent on phosphorylation within the activation loop (also referred to as the “activation segment” or “T-loop” or αT-helix). Phosphorylation within the activation loop fixes the segment into an “active” conformation, thereby forming the primary binding site for the target peptide region of a substrate–protein and stabilizing the active conformation of the catalytic residues.^49^ Many kinases, however, require phosphorylation by a different upstream kinase that specifically phosphorylates the downstream enzyme as a legitimate substrate.^50^ Some protein kinases are activated via an intracellular mechanism (*cis*-mechanism) coupled with protein folding.^51,52^ Glycogen synthase kinase 3β (GSK3β) autophosphorylates Y216 as an intracellular tyrosine kinase in the cascade to becoming the mature enzyme, a Ser/Thr kinase.^51^ The p21-activated kinases (PAKs) are Ser/Thr kinases inactivated by blocking the active site of the kinase domain with an N-terminal regulatory domain. The activation by autophosphorylation in PAKs occurs via dimerization in which two kinase domains are arranged face-to-face and interact through a surface on the large lobe of the kinase domain that is exposed upon release of the autoinhibitory domain.^52^

Based on sequence homology and literature, the CaMK1 group of kinases shows conserved regulation up to the end of the calmodulin binding domain (Figure S5). Differences arise in the C-termini, which probably house the sites of specificity and functionality as defined by the high levels in serine, threonine, and cysteine residues. Homology modeling was used in the discussion and interpretation of the data to assist in drawing intuitively relevant conclusions in developing a new model for the activation and functionality of kinases based on the phosphorylation and cysteine cross-linking data obtained.

The major functional PTMs identified, resulting from the synergistic effect of ATP, redox potential, and Ca^2+^/calmodulin, play out in the following manner. CaMK1δ can select inhibition either by cysteine or by phosphate cross-linking via the activation loop binding to the autoinhibition domain. The phosphate loop-linking, cysteine cross-linking and loop-linking, and tyrosine phosphorylation identified serve as an array of snapshots of the changes in the structure of CaMK1δ in response to the activation by the second messengers that regulate it, namely Ca^2+^/calmodulin, ATP, and redox potential (defined by the presence of oxidizing and reducing agents and H_2_O_2_ as a second messenger).

Domains located at the C-terminus of the activation loop are specific to each kinase family. The data show that Ser/Thr kinases undergo directed, energy-driven conformational changes in response to distinct small-molecule second messengers. During phosphoryl transfer, these structural shifts can also affect associated substrate proteins, meaning that Ser/Thr kinases both phosphorylate substrates and drive their conformational change. This model explains the extensive C-terminal phosphorylation of CaMK1δ and the observed pattern of PTM distribution, including a final C-terminal segment that remained unmodified under the tested conditions and likely represents a distinct regulatory domain responsive to conditions not captured in this study.

## Supplementary Material



## Data Availability

The mass spectrometry raw data included in this paper have been deposited to the ProteomeXchange Consortium via the MassIVE partner repository with the data set identifier PXD069258.^36,53^

## Notes

The use of ATP and DTT for monomer and dimer selection; autophosphorylation and enzyme assay; additional structural selection; tryptic digestion and β-elimination-Michael’s addition (BEMAD); in-gel tryptic digestion; molecular shifts induced by ATP and calmodulin (Table S1); summary of identified phosphate cross-linking and loop-linking spectra using pLink identification, from two distinct experiments (Table S2A and B); identified phosphate loops dominating the activation loop and the C-terminus (Table S3A and B); identification of BEMAD modification sites by phosphorylation, aminoethylthiolation, and/or phosphoric acid loss (Table S4); single-site phosphorylations enumerated at pre- and post-BEMAD treatment (Table S5); site-specific dehydration (−18 Da) modification on both phosphorylation and BEMAD reactions (Table S6); specific site −18 Da only occurring under BEMAD reactions interpreted as a phosphoric acid loss (Table S7); cysteine cross-linking from dialysis conditions used for conformation selection (Table S8); effect of *in vitro* dialysis conditions on cysteine loop formation (Table S9); cysteine cross-linking in redox bands (Figure 6) (Table S10); identification of tyrosine phosphorylation sites in CaMK1δ (Table S11A and B); raw file list for pre- and post-BEMAD experiments (Table S12); raw files for redox band reactions (Table S13); FragPipe analysis for the mass shifts indicative of phosphate loop-linking and cross-linking (Table S14); the mechanism for the proposed phosphate cross-linking (Figure S1); fragmentation of the activation loop and αC region from BEMAD-treated samples (Figure S2); fragmentation of the catalytic region and the C-terminal (Figure S3); effect of CaMK1δ structural isoform selection on kinase enzyme activity (Figure S4); sequence alignment of the CAMK1 group (Figure S5); CaMK1δ homology models, based on CaMK1α (Figure S6); CaMK1δ homology models based on CaMK1α and Chk2 (Figure S7); initiation of the structural rearrangement in the monomer of CaMK1δ via the phosphorylation of Thr184 (Figure S8); the role of Tyr198 in the structural rearrangement of CaMK1δ in the *trans*-autophosphorylation configuration (Figure S9); schematic of the sample preparation leading to kinase assays (Figure S10); nondenaturing PAGE and Western blot (Figure S11) (PDF)
