# Supplementary material for: Proteomic Snapshots of Structural Cross-Linking Rearrangements in Ca2+ /Calmodulin-Dependent Kinase-1-Delta Associated with Its Regulation by ATP, Ca2+ /Calmodulin, and Reduction Potential
Source: J Proteome Res. 2026 Mar 16;25(4):1914–28. doi: 10.1021/acs.jproteome.5c00981 (PMC13054875; doi:10.1021/acs.jproteome.5c00981)
Supplement: Supplementary file 1 [file pr5c00981_si_001.pdf]

SUPPORTING INFORMATION

Proteomic snapshots of structural cross-linking rearrangements in Ca^{2+}/calmodulin-dependent kinase-1-delta associated with its regulation by ATP, Ca^{2+}/calmodulin and reduction potential.

Lutho Mbabala[1], Ndivhuwo O. Tshililo[1], Mare Vlok[2], Iolanda Vendrell[3], Roman Fischer[3], David L. Tabb[4], Catharine A. Trieber[5], Trixie Rae C. Adra[5], Michael Overduin[5], Sam Butterworth[6], and Colin P. Kenyon[1*]

1. South African Medical Research Council Centre for Tuberculosis Research, Division of Molecular Biology and Human Genetics, Faculty of Medicine and Health Sciences, Stellenbosch University, Cape Town 7505, South Africa
2. Trace Labs, Camdeboo Str, Belville 7505, Cape Town, South Africa
3. Target Discovery Institute, Centre for Medicines Discovery, Nuffield Department of Medicine, University of Oxford, Oxford OX3 7FZ, UK
4. European Research Institute for the Biology of Ageing, University Medical Center of Groningen, Groningen 9713 AV, Netherlands
5. Department of Biochemistry, Faculty of Medicine and Dentistry, University of Alberta, Edmonton T6G 2H7, Canada
6. Division of Pharmacy and Optometry, School of Health Sciences, Manchester Academic Health Sciences Centre, University of Manchester, Manchester M13 9PL, UK

TABLE OF CONTENTS

Detailed methods

The use of ATP and DTT for monomer and dimer selection; Autophosphorylation and enzyme assay.

Additional structural selection

Tryptic digestion and β-Elimination-Michael's Addition (BEMAD); In-gel tryptic digestion. **Supplementary Tables**

Molecular shifts induced by ATP and Calmodulin (Table S1)

Summary of identified phosphate cross-linking and loop-linking spectra using pLink identification, from two distinct experiments (Table S2)

Identified phosphate loops dominating the activation loop and the C-terminus (Table S3)

Identification of BEMAD modification sites by phosphorylation, aminoethylthiolation and/or phosphoric acid loss (Table S4)

Single site phosphorylations enumerated at pre- and post-BEMAD treatment (Table S5)
Site specific dehydration (-18 Da) modification on both phosphorylation and BEMAD reactions (Table S6)

Specific site -18 Da only occurring under BEMAD reactions interpreted as a phosphoric acid loss (Table S7)

Cysteine cross-linking from dialysis conditions used for conformation selection (Table S8)

Effect of *in-vitro* dialysis conditions on cysteine-loop formation (Table S9)

Cysteine cross-linking in redox bands (Table S10)

Identification of tyrosine phosphorylation sites in CaMK1δ (Table S11)

Raw file list for pre and post BEMAD experiments (Table S12)

Raw files for redox bands reactions (Table S13)

FragPipe analysis for the mass sifts indicative of phosphate loop-linking and cross-linking. (Table S14)

Supplementary Figures

The mechanism for the proposed phosphate cross-linking (Figure S1)

Fragmentation of the activation loop and αC region from BEMAD treated samples (Figure S2)

Fragmentation of the catalytic region and the C-terminal (Figure S3)

Effect of CaMK1δ structural isoform selection on kinase enzyme activity (Figure S4)

Sequence alignment of the CAMK1 group (Figure S5)

A CaMK1δ homology models, based on CaMK1α (Figure S6)

CaMK1δ homology models based on the CaMK1α (Figure S7)

Initiation of the structural rearrangement in the monomer of CaMK1δ via the phosphorylation of Thr184 (Figure S8)

The role of Tyr198 in the structural re-arrangement of CaMK1δ in the trans-autophosphorylation configuration (Figure S9)

Schematic of the sample preparation leading to kinase assays (Figure S10)

Non-denaturing PAGE and Western blot (Figure S11).

DETAILED METHODS

Reagents.

Reagents used were purchased from Merck Life Sciences (Pty) Ltd, South Africa unless otherwise indicated.

The use of ATP and DTT for monomer and dimer selection

Several steps were taken to mitigate the shortcomings of protein purification in *E. coli* after nickel-column purification. The His_6-tag was cleaved off with TEV for improved folding, and the aberrant phosphorylations that occur in *E. coli* during production were removed by lambda phosphatase. Buffers used in the purification contained DTT to limit misfolding via cysteine bonds. We used buffers, with or without DTT and ATP for conformation selection to achieve the dimers induced by either phosphate and/or cysteine.Dialysis of the protein was performed in two buffers (monomer and dimer) to skew the conformational landscape. The two buffers consisted of the same base composition: 50 mM HEPES, 0.02% NaN_3, 500 mM NaCl, and pH 7.5 in distilled water. The difference between the monomer and dimer buffers was in the regulatory molecules; in the monomer buffer ATP and calmodulin were excluded and only dithiothreitol (0.5 mM DTT) was added [-ATP +DTT -Cam]; in the dimer buffer, only ATP was added [+ATP -DTT -Cam]. Proteins were dialysed for 48 hours at 4°C.

Autophosphorylation and enzyme assay

The enzyme assays were prepared in two phases; first, the autophosphorylation step and second, the kinase activity assay by phosphorylating a substrate peptide, Camtide (KKALRRQETVDAL) (Altabioscience, UK). The assay buffer comprised 50 mM HEPES at pH 7.5, 10 mM MgCl, NaCl 150 mM, 1 mM ATP, 50 µM $CaCl_2$ and calmodulin was added to the dimer reactions at a 2:1 concentration ratio with the CaMK1δ. Autophosphorylation was run for 60 min in a 100 µL and then 2 µL of autophosphorylation reaction was added to the enzyme activity assay (300 µL). No extra calmodulin was added in the kinase assay stage. The Camtide assay concentration was at 500 µM. This assay ran for 180 min and was stopped by the addition of 30 µL of 500 mM EDTA (45 mM). A volume of the autophosphorylation reaction was also analysed using 10% native PAGE gels to assess structural conformation obtained during the autophosphorylation, **Figure S.**

The HPLC based kinase enzyme activity was done with a C18 Luna reverse phase column. The mobile phase comprised 10.5 g KH_2PO_4 in filtered (0.8 μm) 1500 mL Milli-Q ultrapure water (18,2 MΩ) plus 420 mL HPLC grade acetonitrile and 5 mL of 1 M tetrabutylammonium dihydrogen phosphate (2.4 mM). HPLC mobile phase flow rate of 1.0 mL/min and the nucleotides were analysed using a UV detector at a wavelength of 259 nm. The specific enzyme activity was calculated on the basis of the ADP formed.

Additional structural selection

The reactions in the autophosphorylation step, i.e. -ATP -CaM, +ATP -CaM, and +ATP +CaM were further refined for use as dialysis buffers for the structural component using mass spectrometry. This resulted in four buffers with the naming based on the hypothetical conformations that we aimed to achieve, 1) Monomer (+DTT -ATP -CaM), 2) Phosphate dimer (+DTT +ATP -CaM), 3) Phosphate-Cysteine dimer (-DTT +ATP -CaM), 4) Phosphate-Cysteine dimer with Calmodulin (-DTT +ATP +CaM). All the buffers consisted of the same base constituents as mentioned earlier for HEPES dialysis in 0. To the respective solutions containing CaMK1δ, ATP was added to a final concentration of 1 mM, the DTT at 0.5 mM, and calmodulin at double the concentration of the protein. Snake-skin dialysis tubing was used (ThermoFisher Scientific, USA). Only the wild-type was used for structural mass spectrometry.

Tryptic digestion and β-Elimination-Michael's Addition (BEMAD)

Due to the lack of reports of phosphate cross-linking in the literature, we decided to have complementary approaches for its detection: 1) looking for the phosphate cross-link using pLink software and 2) by chemically targeted identification using ß-elimination and Michael's addition (BEMAD). In the BEMAD approach a nucleophilic addition of the 2-aminoethylthiol (AET) yields an aminoethylcysteine thereby locating and tagging the phosphate site. On analysis phosphate cross-linking data was correlated with the BEMAD.

Tryptic digestion was performed using MagReSyn® HILIC beads following the manufacturer's protocol (ReSyn Biosciences, SA). A denaturation step consisting of resuspending 50 µg of sample in 50 µL of 8M Urea, 1% SDS, 5 mM TCEP, pH 7.8 HEPES buffer. Samples were treated with S-methyl methanethiosulfonate (MMTS) (Merck, Germany) to give a ß-methylthiolation tag to help decipher whether the cross-linking was due to phosphate cross-linking and not a disulphide bond. Peptides derived from the four

reaction mixtures were divided in two. The first half was analysed in the standard peptide search looking for phosphorylations and phosphate cross-linking using the pLink software. The second half was treated with β-elimination as a complementary approach for detecting phosphate cross-linking. All solutions were made in doubly deionized water at 18 megaohm from the Milli-Q system (Millipore Corp., USA). Peptides were bound onto an in-house C18 'ZipTip' (C18 membrane matrix, Merck, Germany). For the BEMAD treatment, $Ba(OH)_2$ (90 mM) in 0.005 % v/v Trifluoro acetic acid (TFA) (Merck, Germany) was flushed 10 times over the C18 Zip-Tip containing the peptides. The tip was enclosed in a 1.5 mL centrifuge tube for 1 hour at 60°C in contact with the $Ba(OH)_2$ solution below and above the C18 bed. The matrix was washed 10 times with 50 μL of 90 mM AET (Merck, Germany) and allowed to stand for 30 min at room temperature. Peptides were then eluted with 50% acetonitrile, dried using a Speedvac (Eppendorf, Germany) and stored at -20 until analysed with LCMS.

In-gel tryptic digestion

Excised bands from non-denaturing PAGE gels, were placed in 1.5 mL Eppendorf tubes and destained until clear with multiple wash and dry steps; washing with 100 mM ammonium bicarbonate containing 50% acetonitrile and drying with 100% acetonitrile. Complete drying was done using a Speed Vac (Eppendorf) set to 35°C for 1 hour, gel pieces were considered dry when they appeared white and small. A volume of 100 μL of 20 mM iodoacetamide was added to the dry gel pieces for alkylation of the reduced/unbound cysteine residues and incubated in the dark for 30 min. The supernatant was discarded, and the gel pieces were washed and dried 3 times with 200 μL of 25 mM ammonium bicarbonate for 15 min with agitation. The supernatant was discarded with each wash and drying cycle. After Speed Vac drying the gel pieces were rehydrated in 50 μL of 0.02 μg/μL trypsin in 25 mM ammonium bicarbonate (working on ice) and incubated on ice for 1 hour. Excess trypsin solution was removed, and 25 mM ammonium bicarbonate was added to cover the gel pieces. The Eppendorf tubes were then wrapped with parafilm to avoid evaporation and were incubated at 37°C for 18 hours of digestion. Peptide extraction began with transferring the supernatant into a fresh 1.5 mL tube (extract 1). Gel tubes were resuspended in additional 50 μL of Milli Q water for 30 min and thereafter removed and pooled with extract 1. The peptide extract

was placed in a Speed Vac set to 45°C until dry. All cross-link data was analysed using pLink 2.0 (Chen *et al.*, 2019).

Supplementary Tables

Table S1 Molecular shifts induced by ATP and Calmodulin (Fig. 1B)

Band position	Average kDa	Band%		
		**Lane 2**	**Lane 3**	**Lane 4**
1	250.00	**0.52**	**1.32**	**3.29**
2	207.96	**1.06**	**1.89**	**7.68**
3	152.49	**2.52**	**3.08**	**10.19**
4	97.55	3.32	4.60	2.64
5	91.50	6.13	5.49	4.83
6	85.73	5.06	2.45	5.70
7	73.13	4.60	1.58	2.93
8	62.68	1.69	0.54	0.46
9	41.46	9.29	29.05	24.38
10	37.76	17.99	15.96	19.83
11	35.28	11.23	17.75	11.18
12	33.82	36.60	16.29	6.89

The table provides relative band as a percentage (ImageLab). Lanes: 2 - dialysis without ATP; lane 3 - dialysis supplemented with 1 mM ATP; lane 4 - dialysis supplemented with ATP and calmodulin. The size exclusion was run in HEPES supplemented with and 1 mM $CaCl_2$ to avoid calcium phosphate precipitate and reduce calmodulin dissociation. Conformation descriptions for the gel are based on theoretical molecular weights. Three distinct domains were obtained in each lane. The higher molecular weight region >100 kDa (band position 1, 2, and 3) and the dimer region at 75-80 kDa on the addition of ATP and calmodulin. Clear band mass shifts are seen in the four monomer bands in the monomer region 36-40 kDa, where the buffers with ATP and calmodulin skew the conformation towards 40 kDa.

Table S2A Summary of identified phosphate cross-linking and loop-linking spectra using pLink identification.

Dialysis condition	Types	Cross-Linked Spectra		Loop-Linked Spectra		Mono-Linked Spectra		Regular Spectra	
Phosphate-Cysteine Dimer with Calmodulin (+ATP, -DTT, +CaM)	Counts	6	5	96	101	11	18	6512	6827
	Percent	0.1	0.1	1.4	1.5	0.2	0.3	98.3	98.2
Phosphate-Cysteine Dimer (+ATP, -DTT, -CaM)	Counts	2	25	79	129	38	12	6866	7704
	Percent	0.029	0.3	1.1	1.6	0.5	0.2	98	97.9
Phosphate Dimer (+ATP, +DTT, -CaM)	Counts	7	2	89	4	38	1	6805	5274
	Percent	0.1	0.038	1.3	0.1	0.5	0.019	98.1	99.9
Monomer (-ATP, +DTT, +CaM)	Counts	1	0	80	128	31	51	6171	7055
	Percent	0.016	0.00	1.3	1.8	0.5	0.7	98.2	97.5

Data searched against 100+ *E.coli* proteins from two sets of experiments. Definitions: Cross-link – A covalent bond formed between two peptides via a cross-linking phosphate. Loop-link – A cross-link that occurs within the same peptide, both reactive sites of the cross-linker bind to a single peptide, making an intrapeptide loop. Mono-link – One reactive site of the cross-linker binds to a peptide, while the other end remains free or is hydrolysed. Regular – no cross-linking reagent bound

Table S2B Summary of identified phosphate cross-linking and loop-linking spectra using identification.

Dialysis condition	Types	Cross-linked spectra	Loop-linked spectra	Mono-linked spectra	Regular spectra
Phosphate-Cysteine Dimer with Calmodulin (+ATP, -DTT, +CaM)	Counts	15	114	45	14 357
	Percent	0.1	0.8	0.3	98.8
Phosphate-Cysteine Dimer (+ATP, -DTT, -CaM)	Counts	21	139	81	13 703
	Percent	0.2	1	0.6	98.3
Phosphate Dimer (+ATP, +DTT, -CaM)	Counts	6	131	54	12 418
	Percent	0.09	1.0	0.4	98.5
Monomer (-ATP, +DTT, +CaM)	Counts	20	132	93	13 411

Percent	0.1	1.0	0.7	98.2

Data searched against the whole *E. coli* proteome, from two sets of experiments. Definitions: Cross-link – A covalent bond formed between two peptides via a cross-linking phosphate. Loop-link – A cross-link that occurs within the same peptide, both reactive sites of the cross-linker bind to a single peptide, making an intrapeptide loop. Mono-link – One reactive site of the cross-linker binds to a peptide, while the other end remains free or is hydrolysed. Regular – no cross-linking reagent bound

Table S3A Identified phosphate loops dominating the activation loop and the C-terminus (100+ *E. coli* proteins)

Dialysis Condition	Identified Peptides	Peptide Mass	Modifications	Best E-value	Total	#spec. exp1	#spec. exp2	Domain
Phosphate-cysteine dimer + Calmodulin	DCLAP**ST**LCSFISSSSGVSGVGAER (6)(7)	2550.11	null	5.72E-19	38	12	26	C-terminal
	GDVM**ST**ACGTPGYVAPEVLAQKPYSK (5)(6)	2748.25	Oxidation[M] (4)	8.53E-60	67	27	40	Activation loop
	GDVMS**T**ACG**T**PGYVAPEVLAQKPYSK (6)(10)	2732.26	null	1.36E-20	3	1	2	Activation loop
	HENIVALEDIYE**S**PNHLYLVMQLV**S**GGELFDR(13)(25)	3779.79	Oxidation[M] (21)	9.81E-38	5	5	0	ß-sheet 4
	LHLG**SS**LDSSNASVSSSLSLASQK(5)(6)	2438.17	null	6.63E-15	5	1	4	C-terminal
Phosphate-cysteine dimer	DCLAP**ST**LCSFISSSSGVSGVGAER (6)(7)	2550.11	null	2.74E-08	51	5	46	C-terminal
	GDVM**ST**ACGTPGYVAPEVLAQKPYSK (5)(6)	2732.25	null	3.24E-39	52	9	43	Activation loop
	GDVMS**T**ACG**T**PGYVAPEVLAQKPYSK (6)(10)	2748.27	Oxidation[M] (4)	4.05E-28	6	1	5	Activation loop
	HENIVALEDIYE**S**PNHLYLVMQLV**S**GGELFDR(13)(25)	3763.79	null	9.00E-12	1	0	1	ß-sheet 4
	KLHLGSSLD**SS**NASVSSSLSLASQK (10)(11)	2566.26	null	1.96E-20	3	0	3	C-terminal
Phosphate dimer	DLKPENLLYY**S**QDEE**S**K(11)(16)	2133.95	null	1.47E-19	3	3	0	E-helix
	GDVM**ST**ACGTPGYVAPEVLAQKPYSK (5)(6)	2732.26	null	1.74E-37	8	8	0	Activation loop
	HENIVALEDIYE**S**PNHLYLVMQLV**S**GGELFDR(13)(25)	3779.79	Oxidation[M] (21)	8.27E-31	3	3	0	ß-sheet 4
Monomer	GDVM**ST**ACGTPGYVAPEVLAQKPYSK (5)(6)	2732.26	null	6.96E-41	49	8	41	Activation loop
	GDVMS**T**ACG**T**PGYVAPEVLAQKPYSK (6)(10)	2732.26	null	4.16E-26	2	0	2	Activation loop
	KIFEFKE**T**LG**T**GAFSEVVLAEEK(8)(11)	2715.28	Phospho[S](15)	6.01E-06	2	0	2	ß-sheet 2

Peptide sequences identified with phosphate-induced loop-linking in CaMK1δ. Dominant phosphate loops were found in the activation loop and C-terminal regions. The loop-links were found in all four dialysis reactions. Peptide mass values, post-translational modifications, and statistical confidence levels - E-values lower than 1.00E-02 were provided. The corresponding protein domain for each identified loop-link is indicated.

Table S3B Identified phosphate loops dominating the activation-loop and the C-terminus (whole *E. coli* proteome).

Dialysis condition	Identified Peptides	Peptide Mass	Modifications	Best E-value	# Spec	Domain
Phosphate-cysteine dimer + Calmodulin	GDVMSTACGTPGYVAPEVLAQKPYSK(5)(6)	2732.26	null	2.957594E-55	44	Activation loop
	GDVMSTACGTPGYVAPEVLAQKPYSK(6)(10)	2732.26	null	4.956043E-17	2	Activation loop
BEMAD	GDVMSTACGTPGYVAPEVLAQKPYSK(5)(6)	2732.26	null	2.957594E-55	36	Activation loop
	GDVMSTACGTPGYVAPEVLAQKPYSK(6)(10)	2732.26	null	4.956043E-17	3	Activation loop
	LHLGSSLDSSNASVSSSLSLASQK(5)(6)	2438.17	null	1.978030E-14	2	C-terminal
Phosphate-Cysteine dimer	GDVMSTACGTPGYVAPEVLAQKPYSK(5)(6)	2732.26	Oxidation[M](4)	1.607781E-48	33	Activation loop
	GDVMSTACGTPGYVAPEVLAQKPYSK(6)(10)	2732.26	null	5.619033E-17	2	Activation loop
	KLHLGSSLDSSNASVSSSLSLASQK(7)(10)	2566.26	null	1.099775E-10	1	C-terminal
	HENIVALEDIYESPNHLYLVMQLVSGGELFDR(13)(25)	3763.79	null	5.182429E-9	1	β-sheet 4
BEMAD	DLKPENLLYYSQDEESK(11)(16)	2133.95	null	7.250964E-5	1	E-helix
Phosphate dimer	GDVMSTACGTPGYVAPEVLAQKPYSK(5)(6)	2732.26	null	4.508854E-55	39	Activation loop
	GDVMSTACGTPGYVAPEVLAQKPYSK(6)(10)	2794.24	Oxidation[M](4); Methylthio[C](8)	1.972015E-26	2	Activation loop
	KLHLGSSLDSSNASVSSSLSLASQK(7)(10)	2566.26	null	9.059104E-13	1	C-terminal
	KLHLGSSLDSSNASVSSSLSLASQK(10)(11)	2566.26	null	2.219205E-10	1	C-terminal
	KLHLGSSLDSSNASVSSSLSLASQK(17)(18)	2566.26	null	1.706808E-16	1	C-terminal

BEMAD	ETLGTGAFSEVVLAEEK(2)(9)	1842.86	null	1.828625E-05	2	β-sheet 2
	DLKPENLLYYSQDEESK(11)(16)	2133.95	null	7.250964E-05	1	E-helix
Monomer	NUN					
BEMAD	NUN					

Peptide sequences identified with phosphate-induced loop-linking in CaMK1δ. The spectra was searched against the whole *E.coli* proteome. Dominant phosphate loops were found in the activation loop and C-terminal regions. The loop-links were found in all four dialysis reactions. Peptide mass values, post-translational modifications, and statistical confidence levels - E-values lower than 1.00E-02 were provided. The corresponding protein domain for each identified loop-link is indicated.

Table S4 Identification of BEMAD modification sites by phosphorylation, aminoethylthiolation and/or phosphoric acid loss

	Analysis for phosphorylation and AET pre-BEMAD treatment										Analysis for phosphorylation and AET post-BEMAD treatment								
		Phosphate PTM frequency				AET PTM frequency						Phosphate PTM frequency				AET PTM frequency			
Peptide	Mod.	MON	DIM noDTT	DIM DTT	DIM CAM	MON	DIM noDTT	DIM DTT	DIM CAM	Charge	Mod.	MON	DIM noDTT	DIM DTT	DIM CAM	MON	DIM noDTT	DIM DTT	DIM CAM
GKESSIENEIAVLR	5S(79.9663)	3	3	4	4	0	0	0	0	3	5S(59.0194)	0	0	0	0	3	5	2	2
GDVMS**T**ACG**T**PGYVAPEVLAQKPY	10T(79.9663), 4M(15.9949), 6T(-18.0106), 8C(45.9877)	4	3	2	0	0	0	0	0	3	10T(59.0194), 4M(15.9949), 6T(-18.0106), 8C(45.9877)	0	0	0	0	9	8	6	3
KLHLG**S**SL	6S(79.9663)	4	8	5	5	0	0	0	0	2	6S(59.0194)	0	0	0	0	5	4	3	3
KLHLG**S**SLDSS	6S(79.9663)	4	4	3	4	0	0	0	0	2	6S(-18.0106)	0	0	0	0	1	1	1	1
KLHLGS**S**LDSSN	7S(79.9663)	2	3	3	2	0	0	0	0	2	7S(59.0194)	0	0	0	0	2	3	2	1
KLHLG**S**SLDSSNA	6S(79.9663)	5	3	3	3	0	0	0	0	2,3	6S(-18.0106)	0	0	0	0	3	5	3	2
KLHLG**S**SLDSSNA	6S(79.9663)	3	2	3	4	0	0	0	0	2,3	6S(59.0194)	0	0	0	0	12	17	18	14
KLHLG**S**SLDSSNAS	6S(79.9663)	2	4	4	7	0	0	0	0	3	6S(59.0194)	0	0	0	0	1	3	1	3
KLHLG**S**SLDSSNASVS	6S(79.9663)	8	8	6	6	0	0	0	0	3	6S(59.0194)	0	0	0	0	2	1	1	2
KLHLG**S**SLDSSNASVS	6S(79.9663)	5	4	6	6	0	0	0	0	3	6S(59.0194)	0	0	0	0	12	9	10	11
KLHLGS**S**LDSSNASVSSSLSL	7S(79.9663)	4	4	4	8	0	0	0	0	3	7S(59.0194)	0	0	0	0	4	6	7	4
KLHLG**S**SLDSSNASVS**S**SLSL	17S(79.9663),	1	2	1	5	0	0	0	0	3,4	17S(59.0194),	0	0	0	0	0	0	0	0

	6S(79.9663)										6S(59.0194)								
KLHLG**S**SLDSSNASVSSSL**S**LASQK	20S(79.9663), 6S(79.9663)	2	3	4	1	0	0	0	0	4,5	20S(59.0194), 6S(59.0194)	0	0	0	0	1	3	3	2
DCLAP**ST**LCSFISSSSGVSGVGAER	2C(45.9877), 6S(79.9663), 7T(79.9663), 9C(45.9877)	6	4	8	9	0	0	0	0	4	2C(45.9877), 6S(59.0194), 7T(59.0194), 9C(45.9877)	0	0	0	0	4	1	1	1

This table shows a semi-tryptic search with the left columns shoiwing the dialysis reactions searched for phosphate and AET modifications, with phosphate only occuring in the pre-BEMAD reactions and the AET in the BEMAD reactions. Dialysis reactions: MON – monomer (+DTT-ATP-CAM), DIM noDTT – phosphate-cysteine dimer (-DTT+ATP-CAM), DIM DTT – phosphate dimer (+DTT+ATP-CAM), and DIM CAM – phosphate-cysteine dimer plus calmodulin (-DTT+ATP+CAM). The N-terminal was least affected by the reaction. There were no significant differences in peptide counts between the reactions. The C-terminal (predominantly disordered domain) showed the highest peptide frequencies. Mass definitions: +79.9663 – phosphorylation; +15.9949 – oxidation; +45.9877 – methylthiolation (MMTS alkylation); -18.0106 (79.9663 + 18.0106 = 97.9769) – phosphoric acid loss; +59.0194 – aminoethylcysteine.

Table S5 Single site phosphorylations enumerated at pre- and post-BEMAD treatment

			Pre-BEMAD Exp1				Post-BEMAD Exp1				Pre-BEMAD Exp2				Post-BEMAD Exp2			
Modified Sequence	Charge	Modification	MON	PCDIM	DIMDTT	DIMCAM	BMON	BPCDIM	BDIMDTT	BDIMCAM	MON	PCDIM	DIMDTT	DIMCAM	BMON	BPCDIM	BDIMDTT	BDIMCAM
ES[79.9663]SIENEIAVLR	2,3	2S(79.9663)	27	24	23	23	11	7	9	10	27	35	34	26	4	3	0	2
ESS[79.9663]IENEIAVLR	2	3S(79.9663)	5	5	6	6	0	0	0	0	1	3	2	1	0	0	0	0
HENIVALEDIY[79.9663]ESPNHLYLVMQLVSGGELFDR	4	11Y(79.9663)	0	2	0	0	2	0	0	0	0	0	0	2	0	0	0	0
HENIVALEDIYESPNHLY[79.9663]LVM[15.9949]QLVSGGELFDR	3,4	18Y(79.9663),21M(15.9949)	0	3	5	15	0	0	0	0	0	0	0	1	0	0	0	0
HENIVALEDIYESPNHLYLVMQLVS[79.9663]GGELFDR	4	25S(79.9663)	0	2	0	1	0	0	0	0	0	0	0	0	0	0	0	0

HENIVALEDIYESPNHLYLVM[15.9949]QLVS[79.9663]GGELFDR	3	21M(15.9949),25S(79.9663)	0	1	2	2	0	0	0	0	0	0	0	0	0	0	0	0
DLKPENLLY[79.9663]YSQDEESK	2,3	9Y(79.9663)	4	2	2	0	1	0	0	0	3	0	4	0	0	0	1	2
DLKPENLLYY[79.9663]SQDEESKIMISDFGLSK	3,4	10Y(79.9663)	0	2	4	1	0	0	0	0	2	1	2	3	0	0	0	0
DLKPENLLYYS[79.9663]QDEESK	2,3	11S(79.9663)	3	4	3	6	0	0	0	0	3	8	3	3	0	0	0	0
DLKPENLLYYSQDEES[79.9663]KIMISDFGLSK	3	16S(79.9663)	0	1	0	3	0	0	0	0	0	1	0	1	0	0	0	0
IMISDFGLS[79.9663]KMEGK	2,3	9S(79.9663)	2	4	5	6	0	0	0	0	0	2	0	3	0	0	0	0
GDVMS[79.9663]TAC[45.9877]GTPGYVAPEVLAQK	2	5S(79.9663), 8C(45.9877)	16	18	16	21	0	0	3	0	20	26	23	27	0	0	0	0
HPWIAGDT[79.9663]ALNK	2	8T(79.9663)	0	0	0	0	0	0	0	0	0	0	0	0	0	1	0	4
KLHLG**S**[79.9663]SLDSSNASVSSSL	2,3	6S(79.9663)	222	209	192	173	46	8	10	24	214	180	172	154	2	6	4	11
KLHLGS**S**[79.9663]LDSSNASVSSSL	2,3	7S(79.9663)	32	64	42	120	8	2	7	8	100	152	48	152	7	7	14	9
KLHLGSSLD**S**[79.9663]SNASVSSSL	2,3	10S(79.9663)	14	17	8	10	3	4	4	6	34	29	24	40	3	2	12	7
KLHLGSSLDS**S**[79.9663]NASVSSSL	2,3	11S(79.9663)	4	13	10	17	3	4	4	4	24	28	25	24	1	1	2	1
KLHLGSSLDSSNA**S**[79.9663]VSSSL	2,3	14S(79.9663)	20	15	23	27	9	7	9	7	50	40	45	39	2	4	7	4
KLHLGSSLDSSNASV**S**[79.9663]SSL	2,3	16S(79.9663)	70	47	56	46	26	21	14	19	80	85	88	68	0	3	0	4
KLHLGSSLDSSNASVS**S**[79.9663]SL	2,3	17S(79.9663)	64	78	61	75	22	8	6	11	44	53	67	44	0	0	0	1
KLHLGSSLDSSNASVSS**S**[79.9663]LS	2,3	18S(79.9663)	4	6	4	11	0	0	0	2	6	9	9	12	0	0	0	0
KLHLGSSLDSSNASVSSSL**S**[79.9663]LASQK	3,4	20S(79.9663)	15	17	19	19	1	2	2	1	11	9	14	7	0	0	0	0
KLHLGSSLDSSNASVSSSLSLA**S**[79.9663]QK	3,4	23S(79.9663)	24	32	27	31	2	2	1	2	12	19	15	15	0	0	0	0

This table shows single phosphorylations occurring across the dialysis reactions: MON – (+DTT-ATP-CAM), DIMDTT – (+DTT+ATP-CAM), PCDIM – (-DTT+ATP-CAM), and DIMCAM – (-DTT+ATP+CAM). The ‘B’ preceeding the dialysis reaction denotes reactions that were treated with BEMAD.

Table S6 Site specific dehydration (-18 Da) modification on both phosphorylation and BEMAD reactions

Modified Sequence	Charge	Modification	MON Exp 1	DIMDTT Exp1	PCDIM Exp1	DIMCam Exp1	BMON Exp1	BDIMDTT Exp1	BPCDIM Exp1	DIMCAM Exp1	MON Exp 2	DIMDTT Exp2	PCDIM Exp2	DIMCAM Exp2	BMON Exp2	BDIMDTT Exp2	BPCDIM Exp2	BDIMCAM Exp2
DAS[-18.0106]TLIR	2	3S(-18.0106)	3	1	2	3	1	2	1	2	0	0	0	0	0	0	0	0
DLKPENLLYYSQDEES[-18.0106]K	2,3	16S(-18.0106)	6	3	6	5	6	4	10	4	10	11	10	12	12	15	8	11
ENGES[-18.0106]SSSWK	2	5S(-18.0106)	25	28	18	21	5	9	5	4	27	37	30	34	5	7	8	9
ES[-18.0106]SIENEIAVLR	2	2S(-18.0106)	17	18	16	20	35	42	50	39	15	16	15	20	28	27	34	28
ESS[-18.0106]IENEIAVLR	2	3S(-18.0106)	2	3	2	0	3	2	2	2	4	6	5	5	4	3	3	4
ET[-18.0106]LGTGAFSEVVLAEEK	2	2T(-18.0106)	21	22	26	29	43	48	45	41	23	24	29	29	66	46	50	51
GKESS[-18.0106]IENEIAVLR	3	5S(-18.0106)	5	6	10	7	6	4	8	5	8	7	5	10	2	3	8	8
HENIVALEDIYESPNHLYLVMQLVS[-18.0106]GGELFDR	3,4	25S(-18.0106)	0	11	11	15	3	2	3	2	3	7	5	19	0	5	0	7
HPWIAGDT[-18.0106]	2	8T(-18.0106)	34	39	37	36	33	34	41	32	72	52	77	53	41	46	61	68
KLHLGSS[-18.0106]LD	2	7S(-18.0106)	1	1	1	2	1	0	0	0	1	2	3	2	0	0	0	0
KLHLGSSLDS[-18.0106]	2	10S(-18.0106)	13	11	10	10	15	16	15	8	10	8	9	7	0	0	0	0
KLHLGSSLDSS[-18.0106]N	2,3	11S(-18.0106)	7	5	6	6	9	12	11	10	5	6	6	4	0	1	0	1
KLHLGSSLDSSNAS[-18.0106]	2,3	14S(-18.0106)	5	8	8	8	5	7	4	3	4	5	3	6	0	0	0	0
MEGKGDVM[15.9949]S[-18.0106]T[59.0194]AC[45.9877]GTPGYVAPEVLAQKPY	3	10T(59.0194),12C(45.9877),8M(15.9949),9S(-18.0106)	2	3	2	5	0	0	0	0	3	4	6	3	0	0	0	0
MEGKGDVMS[-18.0106]TAC[45.9877]GTPGYVAPEVLAQKPY	2,3,4	12C(45.9877),9S(-18.0106)	19	20	25	4	0	0	0	0	13	11	11	4	0	0	0	0
M[15.9949]EGKGDVMS[-18.0106]TAC[45.9877]GTPGYVAPEVLAQKPYSK	4	12C(45.9877),1M(15.9949),9S(-18.0106)	3	2	2	1	0	0	0	0	3	4	7	4	0	0	0	0

MEGKGDVMS[-18.0106]TAC[45.9877]GTPGYVAPEVLAQKPYSK	3,4,5	12C(45.9877),9S(-18.0106)	22	15	13	2	0	0	0	0	19	10	4	1	0	0	0	0
NIHESVS[-18.0106]AQIR	2,3	7S(-18.0106)	18	24	27	27	29	31	28	28	32	29	38	42	42	32	54	48
NIHES[-18.0106]VSAQIR	2,3	5S(-18.0106)	15	12	13	15	19	18	18	15	37	32	30	32	40	31	43	36
PENLLYYSQDEESKIMIS[-18.0106]	2,3	18S(-18.0106)	7	7	5	5	1	3	1	1	8	7	8	2	7	51	0	0
YT[-18.0106]C[45.9877]EQAAR	2	2T(-18.0106),3C(45.9877)	6	9	3	6	0	0	0	0	4	5	6	7	0	0	0	0

This table shows dehydration sites across the dialysis reactions: MON – (+DTT-ATP-CAM), DIMDTT – (+DTT+ATP-CAM), PCDIM – (-DTT+ATP-CAM), and DIMCAM – (-DTT+ATP+CAM).a -18 Da occurring on both phosphorylation and BEMAD reaction was interpreted as a loss water.

Table S7 Specific site -18 Da only occurring under BEMAD reactions interpreted as a phosphoric acid loss.

Modified Sequence	Charge	Modification	MON Exp 1	DIMDTT Exp1	PCDIM Exp1	DIMCam Exp1	BMON Exp1	BDIMDTT Exp1	BPCDIM Exp1	DIMCAM Exp1	MON Exp2	DIMDTT Exp2	PCDIM Exp2	DIMCAM Exp2	BMON Exp2	BDIMDTT Exp2	BPCDIM Exp2	BDIMCAM Exp2
ETLGT[-18.0106]GAFSEVVLAEEK	2	5T(-18.0106)	0	0	0	0	2	3	2	3	1	1	0	1	4	6	6	4
GDVM[15.9949]S[-18.0106]T[59.0194]AC[45.9877]GTPGYVAPEVLAQKPY	2,3,4	4M(15.9949),5S(-18.0106),6T(59.0194),8C(45.9877)	0	0	0	0	12	23	14	14	2	0	0	0	0	0	0	4
GKES[-18.0106]SIENEIAVLR	2,3	4S(-18.0106)	0	2	0	1	10	9	10	7	0	0	0	1	22	16	30	27
HPWIAGDT[-18.0106]ALN	2	8T(-18.0106)	1	0	0	0	4	3	7	5	1	1	1	0	4	6	12	9
KLHLGS[-18.0106]SLDSSNASVSSSLSLASQK	3,4	6S(-18.0106)	2	3	2	1	23	27	22	16	0	0	0	0	34	46	44	44
KLHLGSSLDS[-18.0106]SNASVSSSLSLASQK	3,4	10S(-18.0106)	0	0	1	0	6	5	1	4	0	0	0	0	4	11	8	25

KLHLGSSLDSSNASVSS[-18.0106]SLSLASQK	3,4	17S(-18.0106)	0	0	0	0	26	21	26	25	0	0	0	0	52	53	47	56
KLHLGSSLDSSNAS[-18.0106]VSSSLSLASQK	3,4	14S(-18.0106)	0	0	1	2	26	20	11	10	1	0	0	0	25	24	26	23
KLHLGSSLDSSNASVS[-18.0106]SSLSLASQK	4	16S(-18.0106)	0	2	0	1	8	6	9	10	0	1	3	2	4	5	9	13
PTT[-18.0106]VTAVHSGSK	2,3	3T(-18.0106)	0	0	0	0	7	5	8	5	0	0	0	0	2	2	1	3
PTTVTAVHS[-18.0106]GSK	2,3	9S(-18.0106)	0	0	0	0	9	6	7	5	0	0	0	0	2	2	2	2
RPRPT[-18.0106]TVTAVHSGSK	3,4	5T(-18.0106)	0	0	0	0	2	2	6	1	0	0	0	0	0	2	0	2
ASVSS[59.0194]SLSLASQK	2,3	5S(59.0194)	0	0	0	0	13	12	13	11	0	0	0	0	1	2	0	2
ASVSSSLSLAS[59.0194]QK	2,3	11S(59.0194)	0	0	0	0	4	4	4	2	0	0	0	0	2	0	0	3
DAS[59.0194]TLIR	2	3S(59.0194)	0	0	0	0	5	5	3	2	0	0	0	0	0	1	2	3
DAST[59.0194]LIR	2	4T(59.0194)	0	0	0	0	3	3	2	3	0	0	0	0	0	0	0	0
DLKPENLLYYSQDEES[59.0194]K	2,3	16S(59.0194)	0	0	0	0	3	4	4	4	0	0	0	0	11	11	11	12
DLKPENLLYYS[59.0194]QDEESK	3	11S(59.0194)	2	2	2	0	7	7	10	9	0	1	0	0	22	22	34	29
ES[59.0194]SIENEIAVLR	2,3	2S(59.0194)	0	0	1	0	30	29	30	25	1	1	0	0	46	38	62	57
ESS[59.0194]IENEIAVLR	2,3	3S(59.0194)	0	0	0	0	13	10	16	8	0	0	0	0	12	18	45	32
KLHLGS[59.0194]SLDSSNASVSSSLSLASQK	3,4,5	6S(59.0194)	0	0	0	0	154	159	158	148	0	0	0	0	16	25	26	40
LHLGSS[59.0194]LDSSNASVSSSLSLASQK	3,4	6S(59.0194)	0	0	0	0	25	25	23	26	0	0	0	0	3	9	2	1
KLHLGSSLDS[59.0194]SNASVSSSL	3	10S(59.0194)	0	0	0	0	42	38	38	41	0	0	0	0	23	50	33	46
LHLGSSLDSS[59.0194]NASVSSSLSLASQK	3,4	10S(59.0194)	0	0	0	0	15	14	13	10	0	0	0	0	6	15	4	6
LHLGSSLDSSNAS[59.0194]VSSSLSLASQK	3,4	13S(59.0194)	0	0	0	0	73	82	67	83	0	0	0	0	3	10	10	11

LHLGSSLDSSNASVS[59.0194]SSLSLASQK	3,4	15S(59.0194)	0	0	0	0	12	11	10	5	0	0	0	0	19	17	19	23
KLHLGSSLDSSNASVSS[59.0194]SLSLASQK	3,4,5	17S(59.0194)	0	0	0	0	60	65	71	44	0	0	0	0	40	40	30	37
HPWIAGDT[59.0194]ALNK	2,3	8T(59.0194)	0	0	0	0	8	5	5	3	0	1	0	0	7	9	15	14
NIHES[59.0194]VSAQIR	2,3	5S(59.0194)	0	0	0	0	11	9	6	6	0	0	0	0	33	21	57	41
NIHESVS[59.0194]AQIR	2,3	7S(59.0194)	0	0	0	0	1	3	4	1	0	0	0	0	1	5	0	4
PT[59.0194]TVTAVHSGSK	2,3	2T(59.0194)	0	0	0	0	2	5	3	2	0	0	0	0	0	0	0	0
PTT[59.0194]VTAVHSGSK	2,3	3T(59.0194)	0	0	0	0	19	18	23	22	0	0	0	0	0	0	0	0
PTTVT[59.0194]AVHSGSK	2,3	5T(59.0194)	0	0	0	0	5	6	7	2	0	0	0	0	0	0	0	0
PTTVTAVHS[59.0194]GSK	2,3	9S(59.0194)	0	0	0	0	16	14	12	13	0	0	0	0	0	0	0	0
RPRPT[59.0194]TVTAVHSGSK	3,4	5T(59.0194)	0	0	0	0	24	29	29	17	0	0	0	0	0	0	0	0
RPRPTTVT[59.0194]AVHSGSK	4	8T(59.0194)	0	0	0	0	1	2	3	2	0	0	0	0	0	0	0	0
SFIS[59.0194]SSSGVSGVGAER	2,3	4S(59.0194)	0	0	0	0	6	7	4	4	0	0	0	0	0	0	7	5
SLS[59.0194]LASQK	2	3S(59.0194)	0	0	0	0	3	2	2	3	0	0	0	0	0	0	0	0
SLSLAS[59.0194]QK	2	6S(59.0194)	0	0	0	0	10	9	7	7	0	0	0	0	0	0	0	0

This table shows phosphoric acid loss across the dialysis reactions: MON – (+DTT-ATP-CAM), DIMDTT – (+DTT+ATP-CAM), PCDIM – (-DTT+ATP-CAM), and DIMCAM – (-DTT+ATP+CAM). This -18 Da modification was not seen on the phosphorylation reactions and seen on the BEMAD, this we interpret as a phosphoric acid loss due to the BEMAD (98 = 18 + 79.99), this occurs on the counter peptide that was involved in cross-linking or loop-linking.

Table S8 Cysteine cross-linking from dialysis conditions used for conformation selection.

Dialysis Condition	Identified Peptide	Peptide Mass	Modifications	Best E-value	Total	#spec exp1	#spec exp2	Domain
Phosphate -cysteine dimer + Calmodlin	GDVMSTACGTPGYVAPEVLAQKPYSK(8)-YTCEQAAR(3)	3607.686	null	1.47E-20	9	9	0	Act. Loop - αI helix
	YTCEQAAR(3)-YTCEQAAR(3)	1879.806	null	3.10E-09	7	3	4	αI helix - αI helix
Phosphate -cysteine dimer	GDVMSTACGTPGYVAPEVLAQKPYSK(8)-YTCEQAAR(3)	3623.681	Oxidation[M] (4)	2.43E-08	16	7	9	Act. Loop - αI helix
	YTCEQAAR(3)-YTCEQAAR(3)	1879.806	null	5.38E-06	6	2	4	αI helix - αI helix
Phosphate dimer	GDVMSTACGTPGYVAPEVLAQKPYSK(8)-YTCEQAAR(3)	3607.686	null	7.27E-10	4	4	0	Act. Loop - αI helix
	YTCEQAAR(3)-YTCEQAAR(3)	1879.806	null	1.54E-07	4	4	0	αI helix - αI helix
Monomer	GDVMSTACGTPGYVAPEVLAQKPYSK(8)-YTCEQAAR(3)	3623.681	Oxidation[M] (4)	3.40E-08	13	6	7	Act. Loop - αI helix
	YTCEQAAR(3)-YTCEQAAR(3)	1879.806	null	8.70E-05	5	3	2	αI helix - αI helix

Dialysis conditions: Phosphate + cysteine dimer + calmodulin (-DTT +ATP +Cam); Phosphate + cysteine dimer – calmodulin (-DTT +ATP –Cam), Phosphate dimer (+DTT + ATP – Cam), Monomer (+DTT –ATP – Cam). Defined criteria: Identified peptides, peptide mass, detected modifications, E-values less than 1.00E-02 were considered significant, total number of spectra, and spectra counts from each of two experiment. The identified cysteine cross-linking involving the activation loop and the αI-helix, providing regulatory insight between the inhibited and active form of the protein.

Table S9 Effect of *in-vitro* dialysis conditions on cysteine-loop formation.

Reaction	Peptide	Peptide Mass	Modification	Best E-value	Total	#spec exp1	#spec exp2	Domain
Phosphate-cysteine dimer + Calmodulin	DCLAPSTLCSFISSSSGVSGVGAER (2)(9)	2428.112	null	2.88E-52	77	16	61	C-term 2
Phosphate-cysteine dimer	DCLAPSTLCSFISSSSGVSGVGAER (2)(9)	2428.112	null	2.88E-38	80	12	68	C-term 2
Phosphate dimer	DCLAPSTLCSFISSSSGVSGVGAER (2)(9)	2428.112	null	4.54E-99	11	11	0	C-term 2
Monomer	DCLAPSTLCSFISSSSGVSGVGAER (2)(9)	2428.112	null	5.48E-109	32	13	19	C-term 2

The reactions present in this table are the dialysis reactions: Phosphate + cysteine dimer + calmodulin (-DTT +ATP +Cam); Phosphate + cysteine dimer – calmodulin (-DTT +ATP –Cam), Phosphate dimer (+DTT + ATP – Cam), Monomer (+DTT –ATP – Cam). These are followed by identified peptides, peptide mass, detected modifications, statistical confidence levels - E-values less than 1.00E-02 were considered

significant, total number of spectra, and spectral counts from each experiment, n=2. The identified cysteine loop-links were only in the C-terminal.

Table S10 Cysteine cross-linking in redox bands (Figure 6).

Reactions	Peptide	Peptide Mass	Modifications	Best E-values	#spectra	Domain
**LANE 4, BAND 1:** Buff +DTT +CaM	GDVMSTACGTPGYVAPEVLAQKPYSK(8)-YTCEQAAR(3)	3623.681	Oxidation[M](4)	6.93E-06	1	Activation loop – αI-helix
	YTCEQAAR(3)-YTCEQAAR(3)	1879.806	null	9.69E-04	4	αI-helix - αI-helix
**LANE 1, BAND 2:** Buff -DTT -CaM	GDVMSTACGTPGYVAPEVLAQKPYSK(8)-YTCEQAAR(3)	3623.681	Oxidation[M](4)	1.31E-08	3	Activation loop – αI-helix
	YTCEQAAR(3)-YTCEQAAR(3)	1879.806	null	1.12E-03	3	αI-helix - αI-helix
**LANE 1, BAND 4:** Buff -DTT -CaM	YTCEQAAR(3)-YTCEQAAR(3)	1879.806	null	1.45E-02	12	αI-helix - αI-helix
**LANE 2, BAND 2:** Buff -DTT +CaM	GDVMSTACGTPGYVAPEVLAQKPYSK(8)-YTCEQAAR(3)	3623.681	Oxidation[M](4)	8.49E-07	18	Activation loop – αI-helix
**LANE 2, BAND 3:** Buff -DTT +CaM	YTCEQAAR(3)-YTCEQAAR(3)	1879.806	null	4.50E-05	8	αI-helix - αI-helix
**LANE 2, BAND 4:** Buff -DTT +CaM	YTCEQAAR(3)-YTCEQAAR(3)	1879.806	null	1.83E-02	3	αI-helix - αI-helix
**LANE 14, BAND 3:** GSSG -DTT +CaM	GDVMSTACGTPGYVAPEVLAQKPYSK(8)-YTCEQAAR(3)	3623.681	Oxidation[M](4)	1.05E-10	9	Activation loop – αI-helix
	YTCEQAAR(3)-YTCEQAAR(3)	1879.806	null	6.46E-07	7	αI-helix - αI-helix
**LANE 14, BAND 4:** GSSG -DTT +CaM	GDVMSTACGTPGYVAPEVLAQKPYSK(8)-YTCEQAAR(3)	3623.681	Oxidation[M](4)	1.78E-14	14	Activation loop – αI-helix
	YTCEQAAR(3)-YTCEQAAR(3)	1879.806	null	8.22E-06	7	αI-helix - αI-helix
**LANE 6, BAND 3:** H2O2 -DTT +CAM	GDVMSTACGTPGYVAPEVLAQKPYSK(8)-YTCEQAAR(3)	3623.681	Oxidation[M](4)	7.19E-09	6	Activation loop – αI-helix
**LANE 6, BAND 4:** H2O2 -DTT +CAM	GDVMSTACGTPGYVAPEVLAQKPYSK(8)-YTCEQAAR(3)	3623.681	Oxidation[M](4)	2.64E-08	14	Activation loop – αI-helix

YTCEQAAR(3)-YTCEQAAR(3)	1879.806	null	1.29E-06	5	αI-helix - αI-helix

This table shows cysteine cross-linking from gel digestions of autophosphorylation run in the absence and presence of H_2O_2 and GSSG. These are followed by identified peptides, peptide mass, detected modifications, statistical confidence levels - E-values less than 1.00E-02 were considered significant, spectral counts from each experiment, n=1. The identified cysteine loop-links were between the activation loop and the αI-helix.

Table S111A Identification of tyrosine phosphorylation sites in CaMK1δ.

Reactions	Peptide	Spectral Count	Modifications	Charge	Domains
Phosphate-cysteine dimer + Calmodulin	DLKPENLLYY**153**SQDEESKIMISDFGLSK	28	**10Y(79.9663)**, 11S(79.9663), 16S(79.9663), 19M(15.9949)	3, 4	Catalytic loop
	HENIVALEDI**Y88**ESPNHL**Y95**LVMQLVSGGELFDR	394	**11Y(79.9663)**, **18Y(79.9663)**, 21M(15.9949), 25S(79.9663)	3, 4	ß4 and ß5
	IKHENIVALEDI**Y88**ESPNHL**Y**LVMQLVSGGELFDR	363	13Y(79.9663), 20Y(79.9663), 23M(15.9949)	3, 4, 5	ß4 and ß5
	QVLDAVY**Y133**LHR	352	8Y(79.9663)	2, 3	αD-helix
Phosphate-cysteine dimer	DLKPENLL**Y152**YSQDEESK	219	11S(79.9663), **9Y(79.9663)**	2, 3	Catalytic loop
	DLKPENLL**Y152Y153**SQDEESKIMISDFGLSK	25	**10Y(79.9663)**, 11S(79.9663), 16S(79.9663), 19M(15.9949), **9Y(79.9663)**	3, 4	Catalytic loop
	HENIVALEDI**Y**ESPNHL**Y95**LVMQLVSGGELFDR	282	**11Y(79.9663)**, 13S(79.9663), **18Y(79.9663)**,	3, 4	ß4 and ß5

			21M(15.9949), 25S(79.9663)		
	IKHENIVALEDI**Y88**ESPNHL **Y95**LVMQLVSGGELFDR	51	13Y(79.9663), 20Y(79.9663), 23M(15.9949)	3, 4	ß4 and ß5
	QVLDAV**Y133**YLHR	470	7Y(79.9663)	2, 3	αD-helix
Phosphate dimer	DLKPENLL**Y152**YSQDEESK	201	11S(79.9663), **9Y(79.9663)**	2, 3	Catalytic loop
	DLKPENLL**Y152Y153**SQDE ESKIMISDFGLSK	22	10Y(79.9663), 19M(15.9949), 9Y(79.9663)	3, 4	Catalytic loop
	HENIVALEDI**Y88**ESPNHL**Y9 5**LVMQLVSGGELFDR	238	**11Y(79.9663)**, **18Y(79.9663)**, 21M(15.9949), 25S(79.9663)	3, 4	ß4 and ß5
	IKHENIVALEDIYESPNHL**Y9 5**LVMQLVSGGELFDR	191	**20Y(79.9663)**, 23M(15.9949)	4, 5	ß4 and ß5
	**Y268** TCEQAAR	36	1Y(79.9663), 2T(79.9663)	2	Autoinhibitory (αI-helix)

Table shows the identification of tyrosine phosphorylation in different domains, spectra searched against 100+ E. coli proteins

.

Table S11B Identification of tyrosine phosphorylation in CaMK1δ.

Peptide Sequence	Assigned Modifications	Charge	Phosphate-cysteine dimer + Calmodulin	Phosphate-cysteine dimer	Phosphate dimer	Monomer
AEYEFDSPYWDDISDSAK	9Y(-18.0106)	2	5	4	4	2
AEYEFDSPYWDDISDSAK	7S(-18.0106),9Y(63.9714)	2,3	18	9	6	5
AEYEFDSPYWDDISDSAK	7S(-18.0106),9Y(79.9663)	2,3	10	9	8	11
DLKPENLLYYSQDEESKIMISDFGLSK	10Y(79.9663)	3,4	1	2	3	1
DLKPENLLYYSQDEESK	9Y(-18.0106)	2,3	5	5	5	2
DLKPENLLYYSQDEESK	10Y(63.9714),9Y(-18.0106)	3	2	3	1	0
DLKPENLLYYSQDEESK	9Y(79.9663)	2,3	1	1	1	1
DLKPENLLYYSQDEESKIMISDFGLSK	9Y(79.9663)	3,4	0	1	3	1
GDVMSTACGTPGYVAPEVLAQKPYSK	13Y(79.9663),4M(15.9949),6T(-18.0106)	3	0	0	1	1
IKHENIVALEDIYESPNHLYLVMQLVSGGELFDR	13Y(-18.0106),23M(15.9949)	4,5	0	0	3	1
PYSKAVDCWSIGVIAYILLCGYPPFYDENDSK	20C(45.9877),2Y(-18.0106)	3,4	11	2	1	0
PYSKAVDCWSIGVIAYILLCGYPPFYDENDSK	2Y(-18.0106),8C(45.9877)	3,4	1	0	2	0

QVLDAVYYLHR	7Y(-18.0106)	2,3	27	28	26	40

Table shows the identification of tyrosine phosphorylation in different domains, spectra searched against the whole *E. coli* proteome.

Table S12 Raw file list for pre and post BEMAD experiments

	Filename	Dialysis Reaction	StartTimeStamp
Experiment 1	FL1193_MSQ1847_20191101_CKenyon_1_DTT-noATP_mono_125ng_OT.mzML	Monomer (-ATP+DTT-Calmodulin)	2019-11-02T05:52:24Z
	FL1193_MSQ1847_20191101_CKenyon_5_DTT-noATP_mono_BEMAD_125ng_OT.mzML		2019-11-03T07:02:48Z
	FL1193_MSQ1847_20191101_CKenyon_2_noDTT_ATP_dimer_125ng_OT.mzML	Phosphate-Cysteine dimer (+ATP-DTT-Calmodulin)	2019-11-02T11:59:30Z
	FL1193_MSQ1847_20191101_CKenyon_6_noDTT_ATP_dimer_BEMAD_125ng_OT.mzML		2019-11-03T12:28:33Z
	FL1193_MSQ1847_20191101_CKenyon_3_DTT_ATP_dimer_125ng_OT.mzML	Phosphate dimer (+ATP+DTT-Calmodulin)	2019-11-02T18:06:36Z
	FL1193_MSQ1847_20191101_CKenyon_7_DTT_ATP_dimer_BEMAD_125ng_OT.mzML		2019-11-03T17:54:07Z
	FL1193_MSQ1847_20191101_CKenyon_4_noDTT_ATP_Cam_125ng_OT.mzML	Phosphate-Cysteine dimer + Calmodulin (+ATP-DTT+Calmodulin)	2019-11-03T00:13:48Z
	FL1193_MSQ1847_20191101_CKenyon_8_noDTT_ATP_Cam_BEMAD_125ng_OT.mzML		2019-11-03T23:19:37Z
Experiment 2	FL1529_MSQ2220_CKenyon_set1_7_Monomer_plDTT_-ATP.mzML	Monomer (-ATP+DTT-Calmodulin)	2023-03-10T22:14:00Z
	FL1529_MSQ2220_CKenyon_set1_8_Monomer_BEMAD_plDTT_-ATP.mzML		2023-03-11T02:30:23Z
	FL1529_MSQ2220_CKenyon_set1_1_Phos-Cys_dimer_bemad_-DTT_plATP.mzML	Phosphate-Cysteine dimer (+ATP-DTT-Calmodulin)	2023-03-11T06:11:14Z
	FL1529_MSQ2220_CKenyon_set1_2_Phos-Cys_dimer_-DTT_plATP.mzML		2023-03-11T10:27:35Z
	FL1529_MSQ2220_CKenyon_set1_3_Phos-Cys_dimer_CAM_BEMAD_-DTT_plATP.mzML	Phosphate-Cysteine dimer + Calmodulin (+ATP-DTT+Calmodulin)	2023-03-11T14:43:57Z
	FL1529_MSQ2220_CKenyon_set1_4_P-C_dimer_CAM_BEMASD_-DTT_plATP.mzML		2023-03-11T19:00:19Z

FL1529_MSQ2220_CKenyon_set1_5_Phosph_dimer_BEMAD_plDTT_plATP.mzML	Phosphate dimer (+ATP+DTT-Calmodulin)	2023-03-11T23:16:42Z
FL1529_MSQ2220_CKenyon_set1_6_Phosph_dimer_plDTT_plATP.mzML		2023-03-12T03:33:07Z

This table shows a semi-tryptic search with the left columns showing the dialysis reactions searched for phosphate and AET modifications, with phosphate only occuring in the pre-BEMAD reactions and the AET in the BEMAD reactions. Dialysis reactions: MON – monomer (+DTT-ATP-CAM), DIM noDTT – phosphate-cysteine dimer (-DTT+ATP-CAM), DIM DTT – phosphate dimer (+DTT+ATP-CAM), and DIM CAM – phosphate-cysteine dimer plus calmodulin (-DTT+ATP+CAM).

Table S13 Raw files for redox bands reactions

Reactions	Raw files
LANE 1, BAND 2: Buff -DTT -CaM	202209_LM_2.raw
LANE 1, BAND 4: Buff -DTT -CaM	202209_LM_4.raw
LANE 2, BAND 3: Buff -DTT +CaM	202209_LM_Buff-D-C.raw
Lane 4, band 1: Buffer + DTT + CaM	202209_LM_Cam_Buff.raw
LANE 2, BAND 2: Buff -DTT +CaM	FL1529_MSQ2220_CKenyon_set2_1_Buf-only_-DTT_Plus_CAM_Band2.raw
LANE 2, BAND 4: Buff -DTT +CaM	FL1529_MSQ2220_CKenyon_set2_2_Buf-only_-DTT_PlusCAM_Band4.raw
LANE 6, BAND 3: H2O2 -DTT +CAM	202209_LM_Cam_H2O2.raw
LANE 6, BAND 4: H2O2 -DTT +CAM	FL1529_MSQ2220_CKenyon_set2_3_H2O2_-DTT_PlusCAM_Band4.raw
LANE 14, BAND 3: GSSG -DTT +CaM	202209_LM_Cam_GSSG.raw
LANE 14, BAND 4: GSSG -DTT +CaM	FL1529_MSQ2220_CKenyon_set2_4_GSSG_-DTT_Plus_CAM_Band4.raw

Table S14 FragPipe analysis for the mass sifts indicative of phosphate loop-linking and cross-linking.

		Phosphate PTMs pre-BEMAD				Phosphate PTMs post-BEMAD				
Peptide	Assigned Modifications	MON	DIM noDTT	DIM DTT	DIM CAM	MON	DIM noDTT	DIM DTT	DIM CAM	Charge
GDVMSTACGTPGYVAPEVLAQKPY	5S(63.9714)	3	2	2	0	9	9	10	15	2, 3
GDVMSTACGTPGYVAPEVLAQKPY	6T(63.9714)	1	4	2	4	7	10	8	6	2, 3
GDVMSTACGTPGYVAPEVLAQKPY	5S(63.9714),8C(57.0215)	0	0	0	0	15	7	10	6	3
GDVMSTACGTPGYVAPEVLAQKPY	10T(63.9714),4M(15.9949),8C(57.0215)	0	0	1	0	9	8	11	5	2, 3
KGDVMSTACGTPGYVAPEVLAQKPYSK	5M(15.9949),6S(62.9636),9C(45.9877)	0	0	0	0	6	5	4	6	3, 4
GDVMSTACGTPGYVAPEVLAQKPY	10T(63.9714),8C(57.0215)	0	0	0	0	4	6	6	2	3
DCLAPSTLCSFISSSSGVSGVGAER	2C(45.9877),6S(63.9714),9C(45.9877)	44	77	58	59	0	0	0	0	5, 6
ASQKDCLAPSTLCSFISSSSGVSGVGAER	13C(57.0215),2S(63.9714),6C(45.9877)	52	70	56	47	0	0	0	0	3, 4
DCLAPSTLCSFISSSSGVSGVGAER	2C(45.9877),6S(63.9714)	22	24	22	11	0	0	0	0	5, 6, 7
DCLAPSTLCSFISSSSGVSGVGAER	2C(57.0215),6S(63.9714)	19	19	14	6	2	2	2	1	2
ASQKDCLAPSTLCSFISSSSGVSGVGAER	10S(63.9714),13C(57.0215),6C(45.9877)	8	15	16	19	0	0	0	0	3, 4
DCLAPSTLCSFISSSSGVSGVGAER	2C(57.0215),6S(62.9636)	10	12	15	9	0	0	0	0	2
DCLAPSTLCSFISSSSGVSGVGAER	6S(63.9714),7T(62.9636)	5	4	6	12	0	0	0	0	2, 3

DCLAPSTLCSFISSSSGVSGVGAER	2C(57.0215),6S(62.9636),7T(79.9663)	3	8	5	11	0	0	0	0	2

The analysis on Fragpipe was done using +62.9 (O=P-O$^-$) and +63.9 single (HO-P-=O). Dialysis reactions: MON – monomer (+DTT-ATP-CAM), DIM noDTT – phosphate-cysteine dimer (-DTT+ATP-CAM), DIM DTT – phosphate dimer (+DTT+ATP-CAM), and DIM CAM – phosphate-cysteine dimer plus calmodulin (-DTT+ATP+CAM).

Supplementary Figures

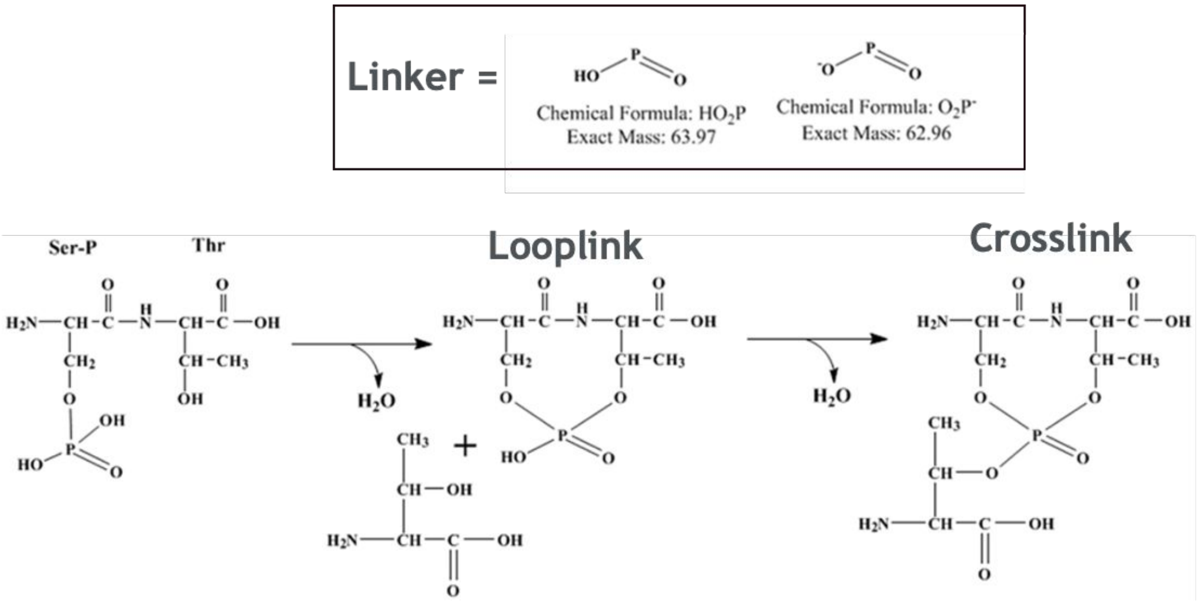

Figure S1 The mechanism for the proposed phosphate cross-linking. Cross-linking via two putative reaction pathways: 1) Loop-link - Ser179 is phosphorylated and Thr184 comes in proximity resulting in a loss of a water molecule between the hydroxyl groups creating a covalent cross-link. The adjacent amino acids on the same peptide would form a loop link while amino acids in two different proteins would form a cross-link. The same reaction could potentially occur to form a three-way crosslink, in which Ser179 cross-links with Thr180 on the same peptide. 2) Cross-link - the reaction starts as a loop between adjacent amino acids Ser179 and Thr180 creating a loop and thereafter a cross-link with the neighbouring activation loop via Thr184.

The spectra presented in **Figure S 2A** and **B** were identified with +3 and +2 charges respectively. Several factors support the validity of these identification (i) the peptide is found in the forward protein reading sequence (ii) it contains both the y and b ion series (iii) the y-ion fragmentation favoured the N-terminal of the proline supporting the proline effect (iv) all major peaks are assigned.

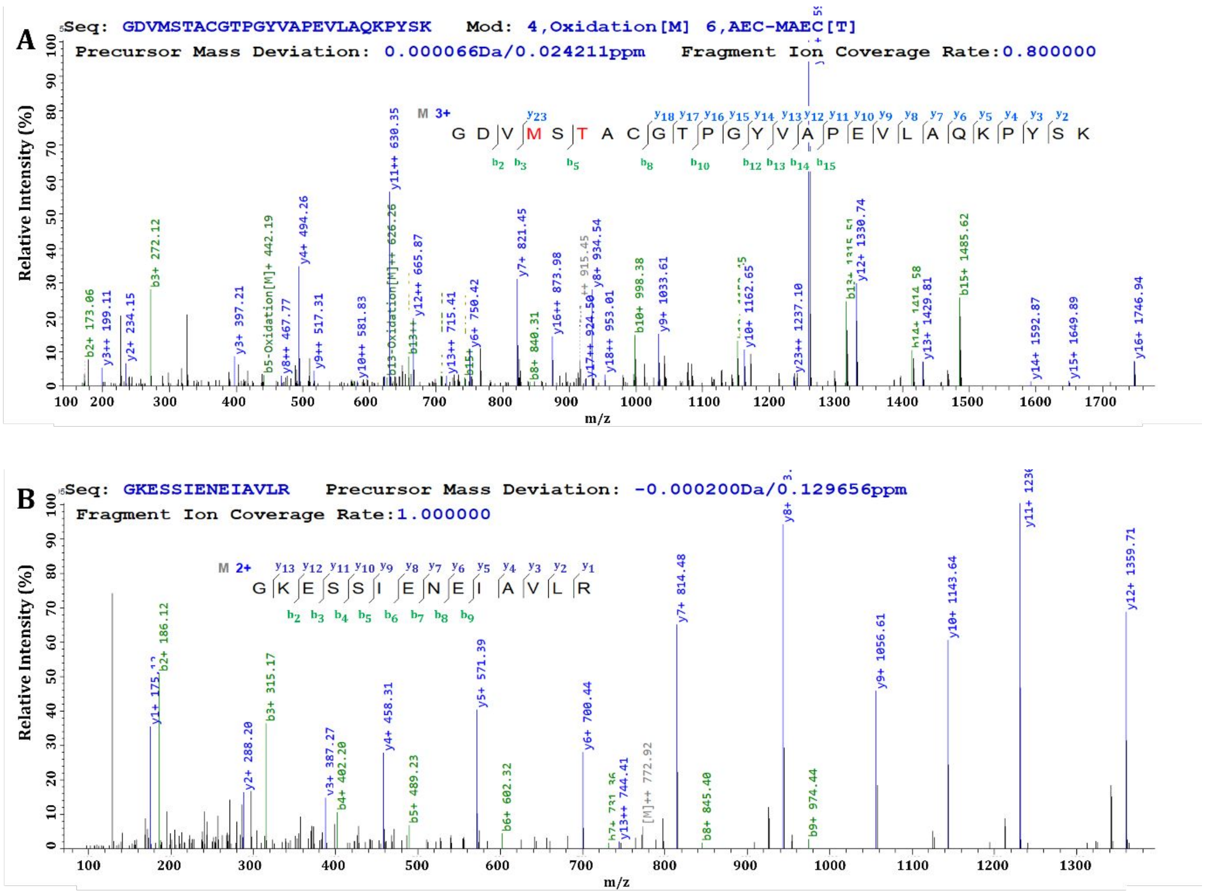

Figure S2 Fragmentation of the activation loop and αC region from BEMAD treated samples. A) Dimer dialysis containing ATP and calmodulin – the activation loop is primarily defined by the y-ion series, with limited b-ions. B) Dimer dialysis containing only ATP - the αC region exhibits strong b- and y-ion coverage.

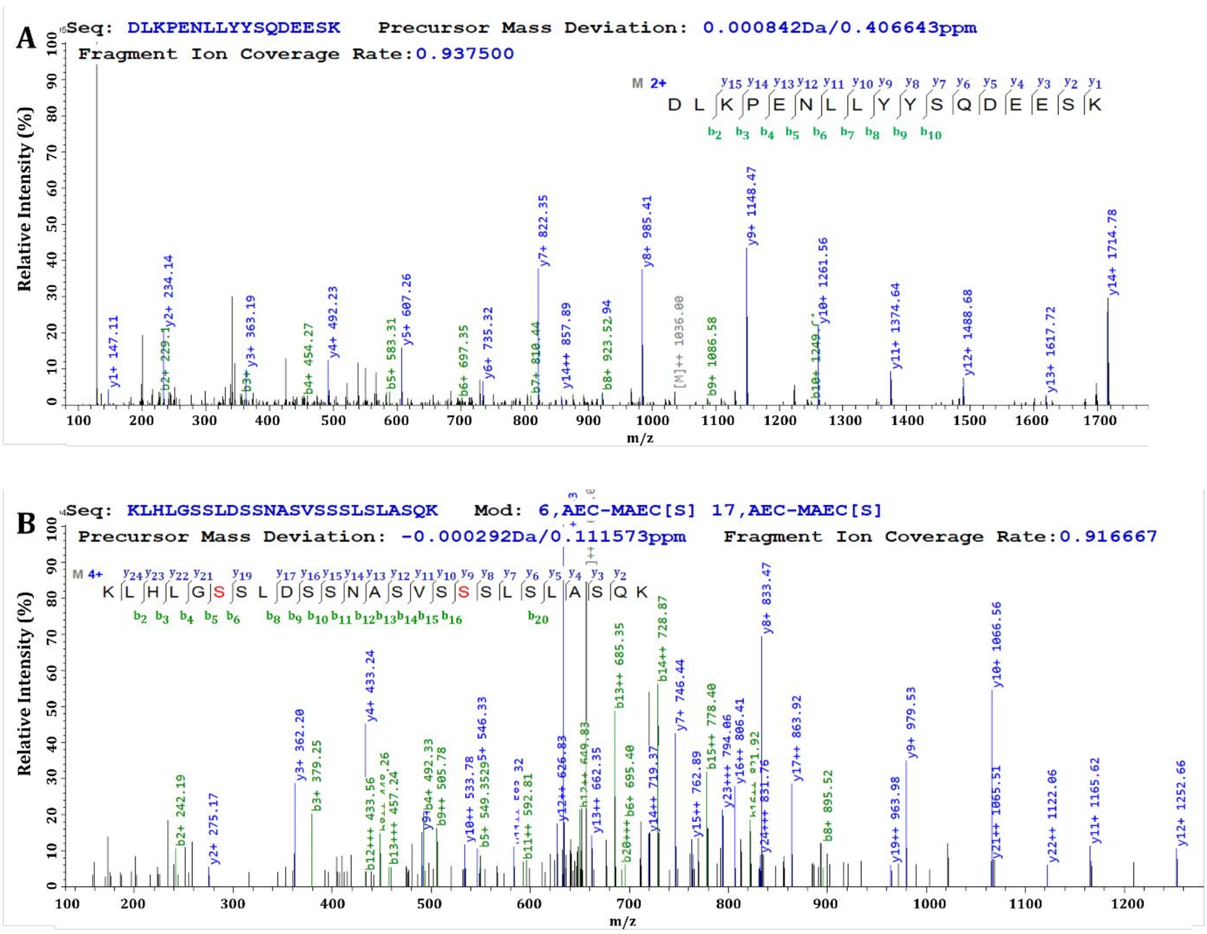

Figure S3 Fragmentation of the catalytic region and the C-terminal. Spectra exhibit key characteristics indicative of true identification based on sequence coverage by b- and y-ions, the proline effect, and major peak assignments.

Error! Reference source not found.

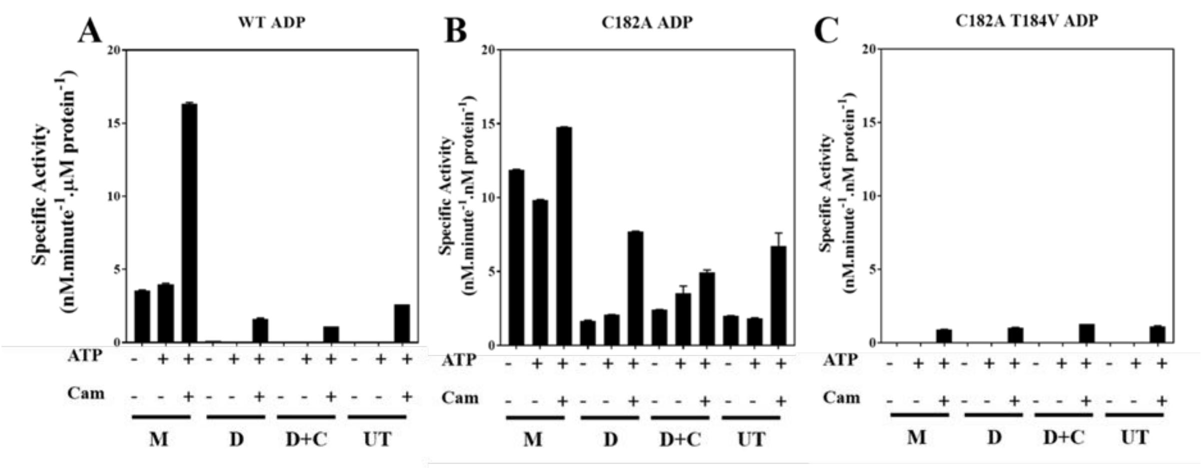

Figure S4 Effect of CaMK1δ structural isoform selection on kinase enzyme activity. Effect of dialysis autophosphorylation conditions on the specific enzyme activity obtained for the WT, C182A and C182A-

T184V mutant enzymes of CaMK1δ. The activity was expressed as specific activity (nM ADP.min^{-1}. mM^{-1} protein). The first three activities contain protein from dialysis without ATP in the presence of DTT selecting for monomers (M - monomer), the second three lanes contain protein from dialysis with ATP in the absence of DTT (D - dimer) selecting for dimer, the third 3 lanes contain protein from the dialysis in the absence of DTT but containing calmodulin (D+C), and the fourth group of three activities contain undialysed protein (UT = untreated protein with no dialysis, untreated for both TEV and phosphatase. The enzyme assay was run for three hours at 37°C, under standard enzyme activity conditions.

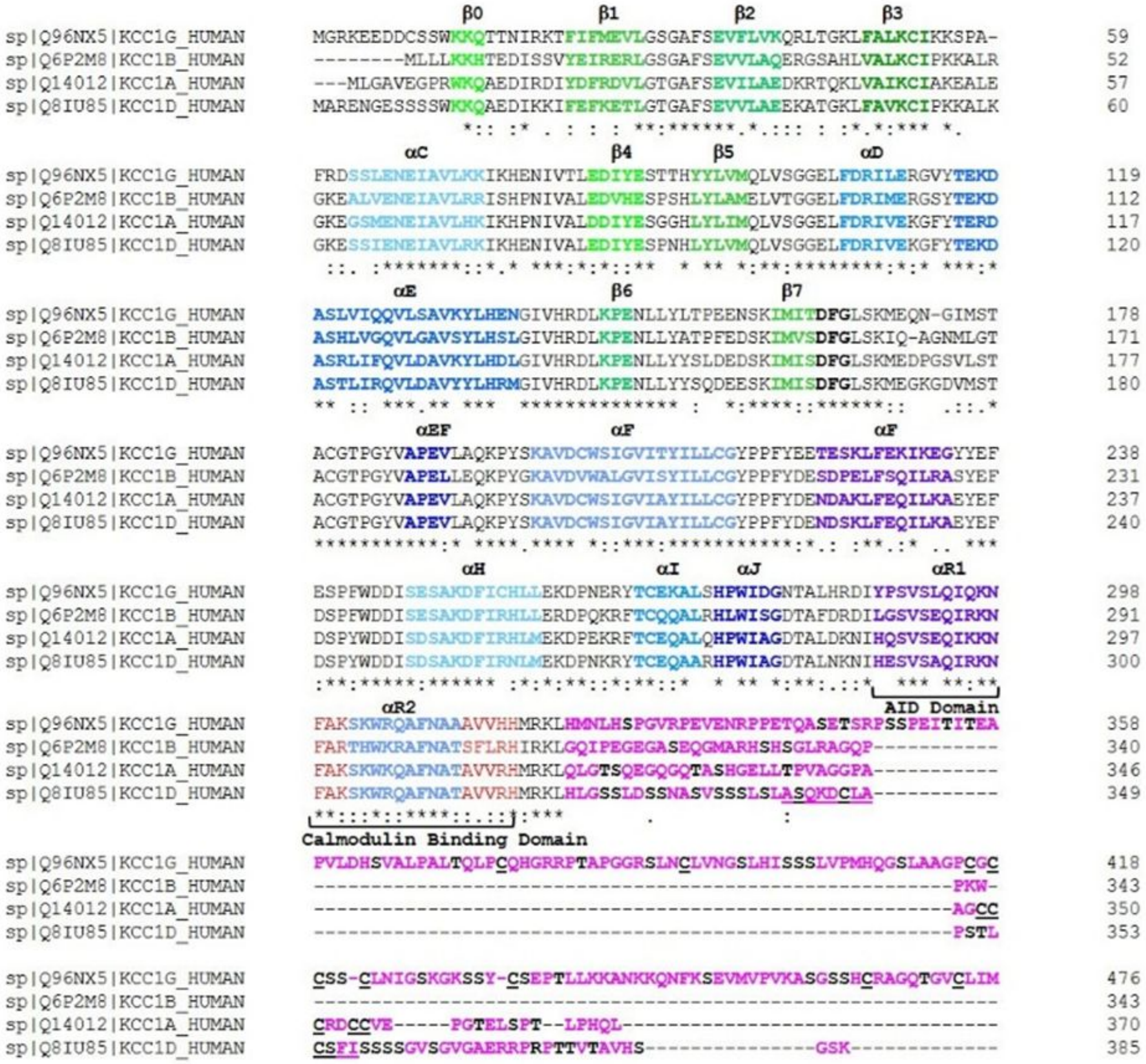

Figure S5 Sequence alignment of the CAMK1 group. The high level of sequence homology up to the end of the calmodulin binding domain as shown by the conserved residues indicated by an asterisk and the conserved functionality indicated by a colon. (% identity 1ß:1γ 58.06 %, 1α:1γ 61.20 %, 1δ:1γ 64.30 %, 1ß:1α 63.56 %, 1ß:1δ 63.27 %, 1α:1δ 73.78 %. (Sequence alignment Clustal W, UniProt(Larkin *et al.*, 2007)) Clustal 0 (1.2.4)

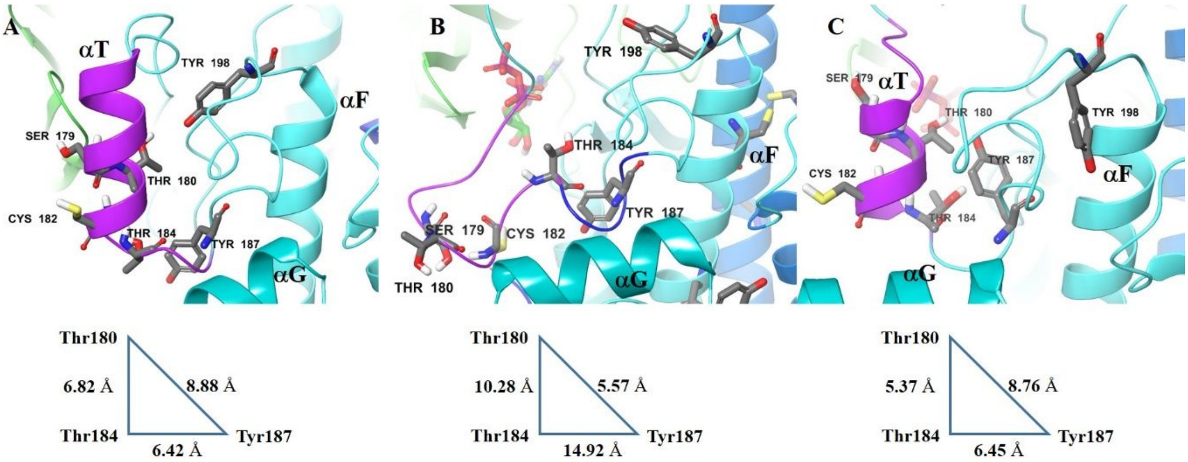

Figure S6 A CaMK1δ homology models, based on CaMK1α (A = 4FGB.pdb, B = 4FG8.pdb, C = 4FG9.pdb). A) apo form (minus ATP) truncated to midway within the αR2 helix, B) ATP complex truncated to the end αR1 helix, C) ATP complex truncated to the end αR2 helix. The triangle below each model shows Cα interatomic distances between Thr180, Thr184 and Tyr187.

A CaMK1δ homology model based on CaMK1α (4FG9.pdb) was compared to Chk2 (2CN5.pdb). Both models share αT helix (G175-T184, GDVMSTACGTP), αR1 and αR2. In Chk2, αT mediates trans-activation via activation loop exchange and shifting of the αR1 helix from occupying the catalytic domain.

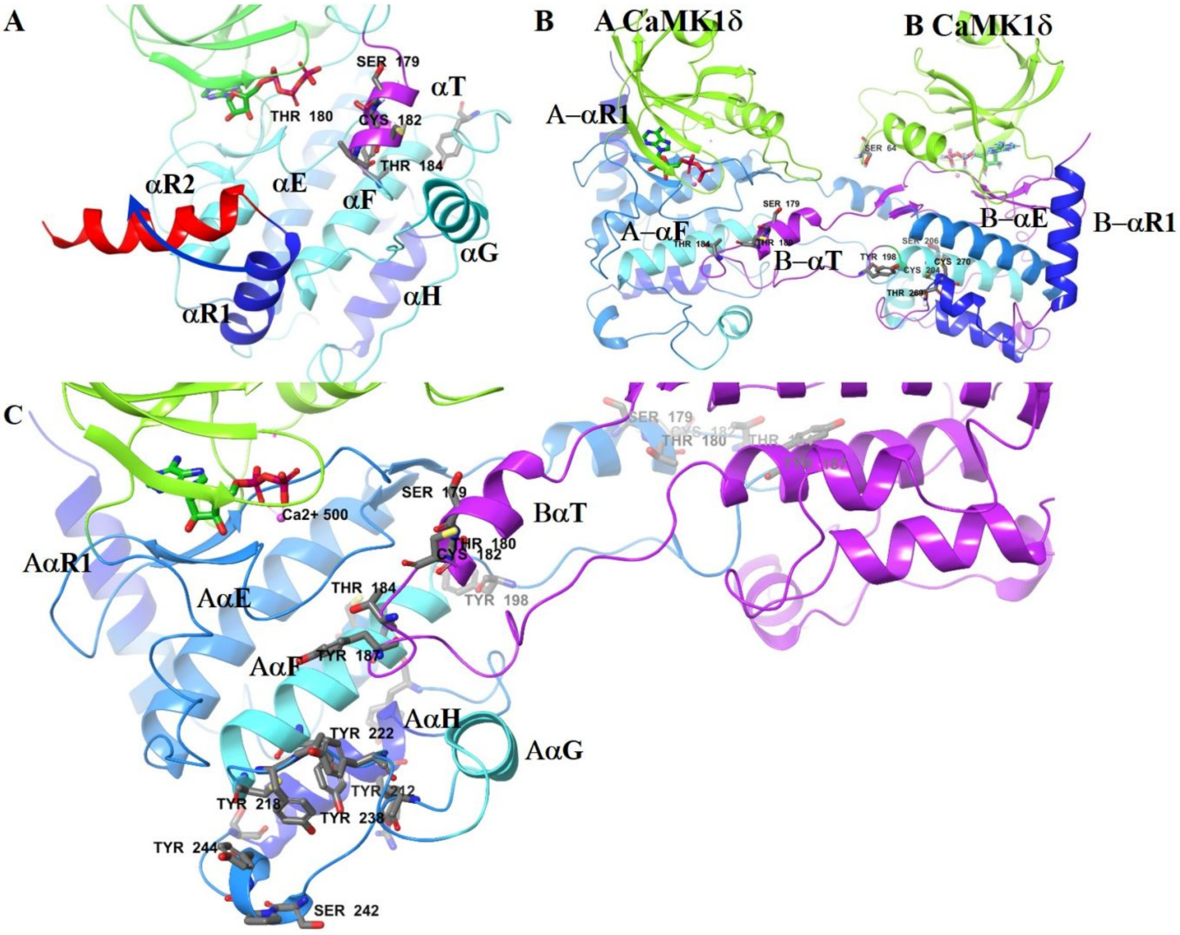

Figure S7 CaMK1δ homology models based on the CaMK1α (4FG9.pdb) and Chk2 (2CN5.pdb) structures. The CaMK1α based structure of CaMK1δ is 315 amino acids in length in complex with ATP (Zha *et al.*, 2012). A) Structure includes helices αR1 and αR2 from the regulatory domain. Arrow indicates direction of rotation of the αR1-helix on activation when compared to structure C. B) CaMK1δ homology model based on the structure of the trans-activation of the DNA-damage signalling protein kinase Chk2 the trans-activation occurring by dimerization and T-loop exchange (2CN5.pdb) (Oliver *et al.*, 2006). C) Trans-activation leads to the rotation of αR1 away from the activation segment (A & C). The phosphorylation of either Ser179, Thr180 and/or Thr184 within the αT helix could lead to phosphoryl transfer to Tyr187.

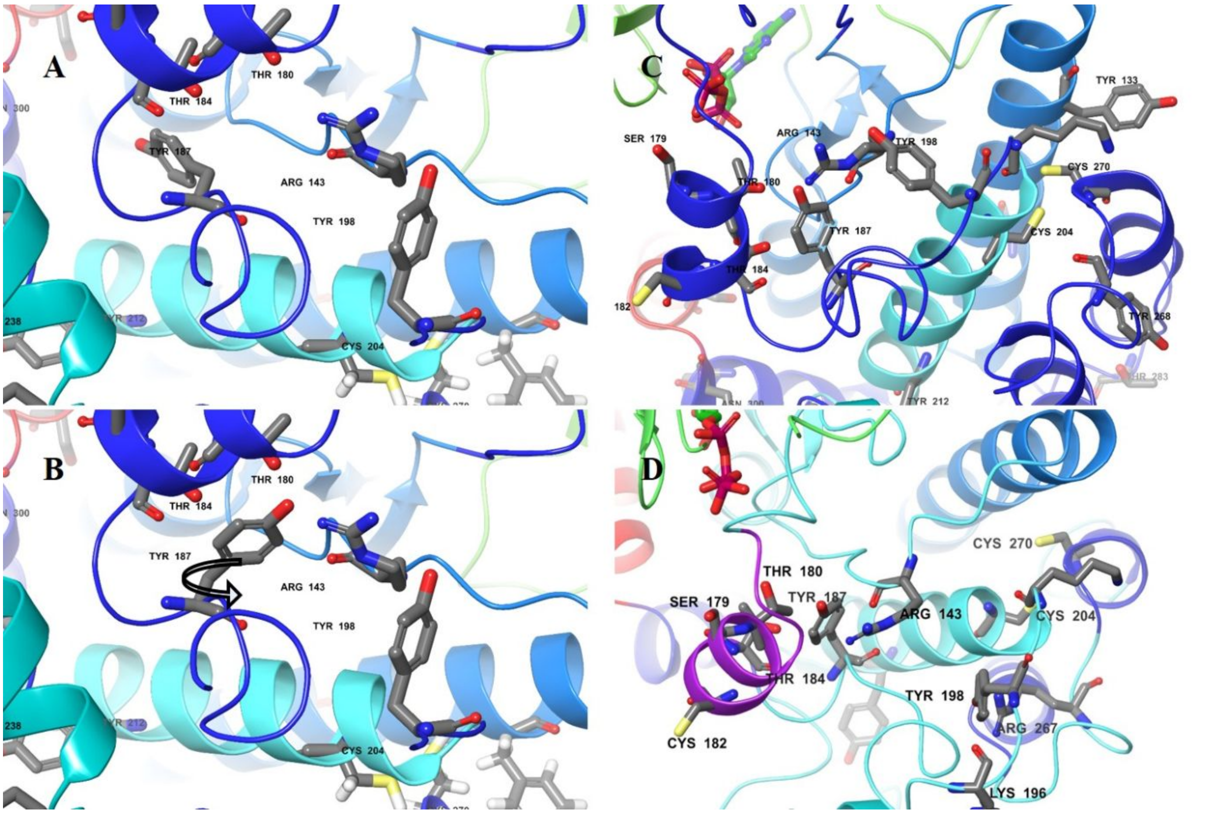

Figure S8 Initiation of the structural rearrangement in the monomer of CaMK1δ via the phosphorylation of Thr184. The transferring of the $PO4^{2-}$ to Tyr187 facilitates an interaction with Tyr198 via Arg143 by the rotation of the Cα-Cß bond of Tyr187 (A & B). Proximity of Tyr198 with Cys204 and Cys270, which are in close enough proximity to form a disulphide bond (C & D). Homology model CaMK1δ using CaMK1α as the template (4FG9.pdb).

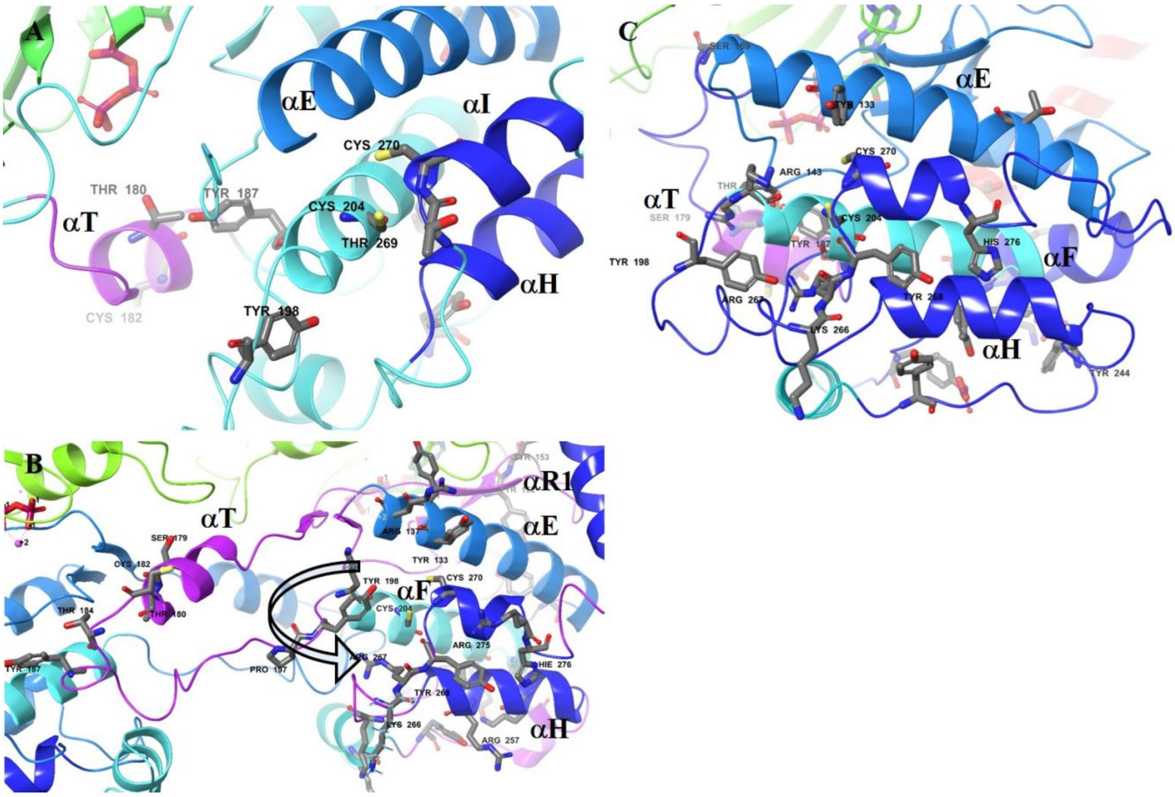

Figure S9 The role of Tyr198 in the structural re-arrangement of CaMK1δ in the trans-autophosphorylation configuration. The two dominant disulphide bonds that are found in the pLink cysteine cross-link analysis are GDVMSTACGTPGYVAPEVLAQKPYSK (Cys182)-YTCEQAAR (Cys270) and AVDCWSIGVIAYILLCGYPPFYDENDSK (Cys204)-YTCEQAAR (270), inhibited and functional forms, respectively. pTyr198 appears to play a putative role in forming both the fully inhibited and functionally active forms of CaMK1δ. pTyr198 interacting with Arg267, Arg275 and His276 could draw the αT helix and the disordered loop region up to Tyr198 into closer proximity folding αT helix into close proximity with αF.

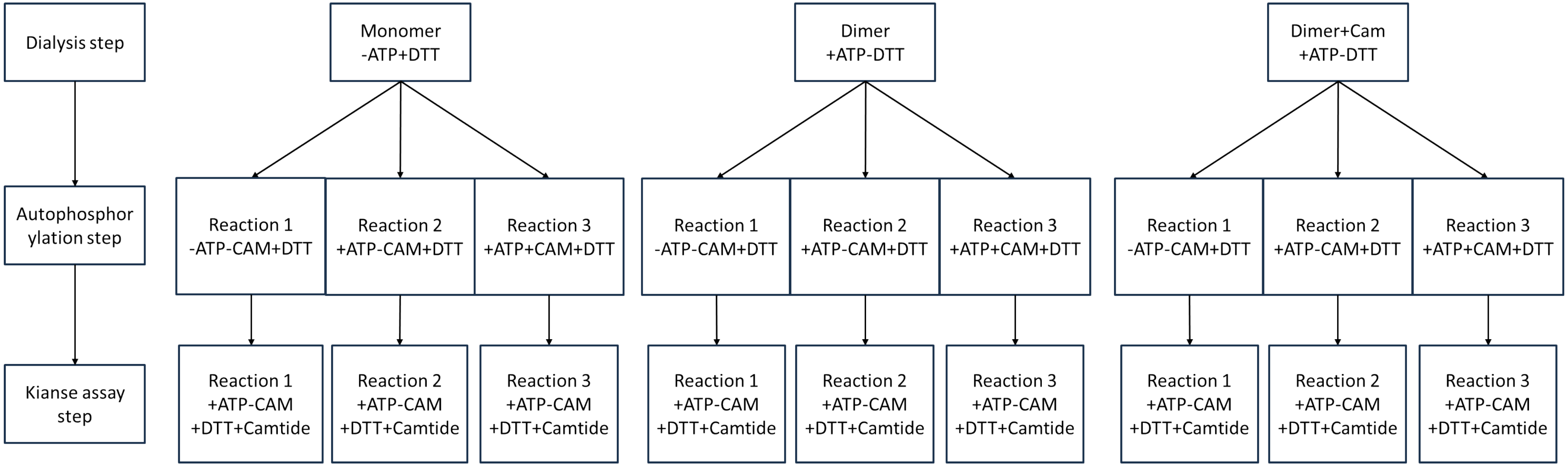

Figure S10 Schematic of the sample preparation leading to kinase assays. Protein was eluted with (50 mM HEPES pH 7.5, 500 mM NaCl, 0.5 mM DTT, 250 mM imidazole, 0.02% NaN_3 and a protease inhibitor cocktail). The His_6-tag was cleaved off in a dialysis containing Tobacco Etch Virus (TEV) protease (1:100) and dialysed in buffer (50 mM HEPES pH 7.5, 500 mM NaCl, 0.5 mM DTT, 0.02% NaN_3) for 24 hours at 4°C. Dephosphorylation of the protein was achieved in dialysis buffer containing lambda phosphatase (50 mM HEPES pH 7.5, 500 mM NaCl, 0.5 mM DTT, 0.02% NaN_3, and 1 mM Mn^{2+}) at 1:100. The protein was further purified using size exclusion (50 mM Na HEPES pH 7.5, 150 mM NaCl, 0.5 mM DTT, 0.02% NaN_3). The pooled protein fractions were then divided into two dialysis reactions for conformation selection: monomer (– ATP – Cam + DTT), and dimer (+ ATP – Cam – DTT). The target dimers were those that were covalently bonded either by disulphide bonds, phosphate-cross-linking or both disulphide bonds and phosphate-cross-linking. The native PAGE gels were used to visualize the conformation equilibrium for the re-assembly of the monomers obtained into dimers. Kinase enzyme assays were conducted by HPLC through measuring the ADP formed during the phosphorylation of Camtide (KKALRRQETVDAL). The HPLC analysis was done with a C18 Luna reverse phase column. The buffer was made up of 10.5 g KH_2PO_4 added to 1500 mL Milli-Q ultrapure water (18,2 MΩ) and filtered with absolute aperture (AA) 0.8µm, 47mm filter paper. HPLC grade acetonitrile of 420 mL and 5 mL of 1 M tetrabutylammonium dihydrogen phosphate (2.4 mM) were added to the filtered KH_2PO_4 buffer, to create the mobile phase (40 mM). The HPLC was run at a mobile phase flow rate of 1.0 mL/min and the nucleotides were analysed using a UV detector at a wavelength of 259 nm. The flush solution was made with 50% HPLC grade acetonitrile in ultrapure water.

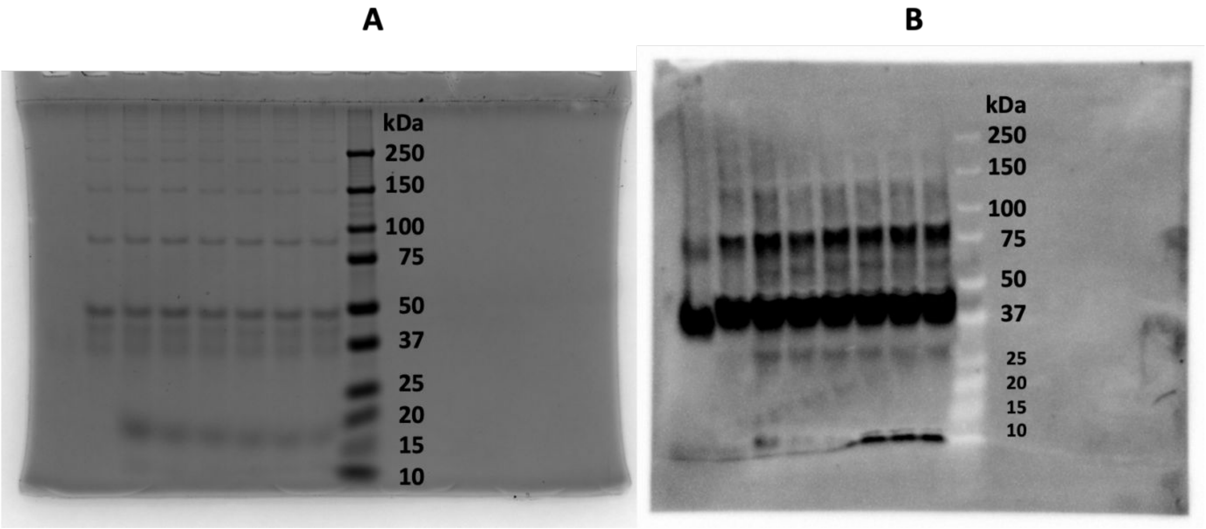

Figure S11 Non-denaturing PAGE and Western blot. A) CaMK1D non-denaturing PAGE, lane 1 – CaMK1D monomer, lane 2 – CaMK1D+ATP, lane 3-8 – CaMK1D+ATP+Calmodulin, lane 9 – protein molecular weight marker. B) Western blot with lanes identical to PAGE.
